# A hybrid ConvNeXt-ViT framework with differential evolution optimization for breast cancer classification

**DOI:** 10.1038/s41598-026-61594-4

**Published:** 2026-07-29

**Authors:** Murdhy A. Aldawsari, Saad Jamhan Aldosari, Atef Ismail, Marwa M. Emam

**Affiliations:** 1 Department of Mathematics, College of Sciences and Humanities, Prince Sattam Bin Abdulaziz University, Al-Kharj, 11942 Saudi Arabia; 2 Physics Department, Al-Azhar University, Asyut, 71524 Egypt; 3 Faculty of Computers and Information, Minia University, Minia, Egypt

**Keywords:** Breast cancer classification, ConvNeXt, Vision transformer, Differential evolution, Multi-head attention, Generative adversarial networks, Cancer, Computational biology and bioinformatics, Engineering, Mathematics and computing

## Abstract

Breast cancer, a leading cause of mortality among women worldwide, necessitates early detection through mammography. Yet, automated classification remains challenging due to class imbalance, limited datasets, and the need for both local and global feature extraction. While convolutional neural networks (CNNs) excel in local feature extraction for mammogram classification, they struggle with long-range contextual dependencies. Conversely, transformer-based models capture global relationships effectively but require large datasets and substantial computational resources, limiting their applicability in medical imaging. To overcome these limitations, we propose DEViTNeXt, a new hybrid framework that synergistically combines ConvNeXt’s convolutional efficiency with Vision Transformer (ViT) attention-based global modeling, enhanced by Differential Evolution (DE) optimization. The framework employs comprehensive preprocessing (Gaussian filtering, CLAHE enhancement) and a hybrid augmentation pipeline that integrates GAN-based synthesis of malignant cases with geometric transformations. Dual-branch feature extraction leverages ConvNeXt for hierarchical local features and ViT for global contextual relationships, with Multi-Head Attention (MHA) refinement dynamically emphasizing diagnostic regions in both branches. A DE-optimized MHA fusion layer adaptively integrates complementary. A composite loss function (Weighted Cross-Entropy + Focal Loss) addresses class imbalance while focusing on complex malignant cases. Extensive experiments on the CBIS-DDSM and MIAS datasets demonstrate DEViTNeXt’s superiority, achieving 99.63% accuracy, 99.45% sensitivity, and 99.55% specificity on CBIS-DDSM under binary (Benign vs. Malignant) classification, and 98.50% accuracy on MIAS (3-class), outperforming state-of-the-art methods.

## Introduction

Breast cancer stands as one of the most prevalent malignancies affecting women globally, posing a significant public health challenge. Recent estimates indicate that millions of new cases emerge each year, leading to substantial mortality rates despite advances in medical care. This disease manifests in diverse forms, with variations in biological behavior that influence treatment outcomes and patient survival^1^. The American Cancer Society reported approximately 281,500 new cases of invasive breast cancer in women and about 3,000 in men in the United States for the year 2022. Of all cancer cases in the U.S., women represent around 30%, with breast cancer accounting for 19% of these diagnoses. Since the mid-2000s, the incidence of breast cancer has been rising by 0.5% each year. On a global scale, the World Health Organization documented over 2.3 million new breast cancer cases in 2020, comprising 11.7% of all new cancer cases and leading to 685,000 deaths among women^2^. Breast cancer develops from the abnormal and uncontrolled proliferation of breast tissue, resulting in tumor formation^3^. Such abnormalities can manifest as micro-calcifications, masses of varying shapes and sizes, asymmetry between breasts, or disruptions in normal tissue architecture^4^.

Early detection and timely treatment of breast cancer have significantly improved five-year survival rates for patients^5^. Breast cancer diagnosis relies on various imaging modalities, including mammograms^6^, breast thermography^7^, magnetic resonance imaging (MRI)^8^, positron emission tomography (PET), computed tomography (CT)^9^, ultrasound^10^, and histopathology^11^. Among these, mammography, utilizing low-dose X-rays, is the most widely adopted screening tool due to its ability to detect abnormalities before palpable lumps develop. Observational studies indicate that mammography screening has reduced breast cancer mortality by up to 40%, underscoring its critical role in early intervention. However, the complexity of mammographic interpretation often leads to diagnostic errors, requiring highly skilled radiologists to ensure accuracy^12^. As the volume of cases increases and image analysis becomes more intricate, manual evaluation by experts remains time-consuming and resource-intensive, placing significant strain on healthcare systems^13^.

Recent advancements in computer vision and deep learning (DL) have revolutionized automated breast cancer diagnosis, enabling advanced computer-aided diagnosis (CAD) systems that support radiologists in early detection and informed clinical decision-making^14–16^. Convolutional neural networks (CNNs) have demonstrated exceptional performance in mammogram analysis by hierarchically learning discriminative spatial features, thereby facilitating the identification of complex tissue structures, pathological patterns, and cancer molecular subtypes, which are critical for personalized treatment strategies^15–17^. Despite their effectiveness, conventional CNNs face limitations, including limited receptive fields that hinder the capture of long-range spatial dependencies and a tendency to overfit on small or imbalanced medical datasets^18^. Consequently, they often fail to model subtle yet clinically significant features, such as microcalcifications and architectural distortions, compromising generalization and clinical reliability for accurate breast cancer diagnosis.

To overcome these challenges, Transformer-based architectures have recently emerged as powerful alternatives in visual recognition tasks due to their ability to model global contextual relationships through self-attention mechanisms^19,20^. In particular, vision Transformers (ViTs) have shown strong potential for medical imaging applications by capturing global and contextual representations from mammograms. ViTs utilize self-attention mechanisms to capture long-range dependencies, enabling effective processing of diverse image resolutions and complex patterns. By treating images as sequences of patches, ViTs learn global contextual information, which is crucial for medical image analysis, where subtle pattern variations are diagnostically significant. These capabilities enhance CAD systems, improving accuracy in applications like breast cancer classification.

However, ViTs lack the intrinsic local inductive biases of CNNs, making them less effective in modeling fine-grained spatial details and requiring large-scale annotated datasets to perform optimally–an impractical condition in medical domains where labeled data are scarce^21^. Recognizing the complementary strengths of CNNs and Transformers, several recent studies have explored hybrid architectures that integrate convolutional feature extractors with self-attention mechanisms. Such approaches aim to balance local feature precision with global contextual understanding, thereby improving diagnostic accuracy and interpretability. Nevertheless, existing hybrid models still face major limitations: (i) they often depend on manually tuned hyperparameters that restrict adaptability; (ii) they rarely address data imbalance and scarcity through advanced augmentation strategies; and (iii) their fusion layers typically fail to fully exploit the complementary nature of features derived from CNN and Transformer branches.

Metaheuristic optimization algorithms have been extensively applied across diverse domains, including medical imaging^22–26^, traffic signal control^27^, and various complex optimization tasks^28,29^, providing flexible and efficient solutions to challenging problems. In the context of deep learning for medical applications, these algorithms excel at automating hyperparameter tuning and enhancing classification performance^30^. Differential Evolution (DE) stands out for its robust global search capabilities, fast convergence, and effective balance between exploration and exploitation, making it ideal for fine-tuning intricate model parameters in breast cancer classification. To ensure the scientific rigor of our study, we carefully selected models based on comprehensive multi-metric evaluations and leveraged DE to optimize hyperparameters, thereby improving diagnostic accuracy and overall model efficacy.

Recent studies have further advanced the field of medical image analysis through sophisticated deep learning techniques. For instance, Abbas et al.^31–34^ proposed various attention-enhanced and mixture-of-experts architectures for Parkinson’s disease, gastrointestinal disorders, and Alzheimer’s diagnosis. Similarly, Attique Khan et al.^35^ introduced a fused self-attention network for cervical cancer classification, while Abdelbaki et al.^36^ developed an efficient hybrid CNN-Transformer model for blood cancer detection. These works highlight the growing effectiveness of attention mechanisms, hybrid architectures, and adaptive expert routing strategies in medical imaging tasks.Moreover, Ullah et al.^37^ proposed a hierarchical deep feature fusion and ensemble learning approach for brain tumor MRI classification. In another study, Ullah et al.^38^ systematically integrated attention mechanisms into multiple CNN backbones (VGG16, ResNet18, InceptionV3, DenseNet121, and EfficientNetB5) and demonstrated consistent performance gains across different medical imaging tasks. Furthermore, Ullah et al.^39^ introduced dense associative networks for anatomically accurate cardiac segmentation using transformer-based architectures.

Motivated by these advancements and their success in handling complex medical patterns, we propose DEViTNeXt, a novel hybrid deep learning framework specifically designed for breast cancer classification from mammograms. Unlike the aforementioned studies that primarily target other medical domains, our work integrates ConvNeXt’s modern convolutional inductive biases with Vision Transformer’s global modeling, further enhanced by Differential Evolution optimization and dynamic multi-head attention fusion. This design specifically addresses the unique challenges of mammographic images, such as subtle microcalcifications, architectural distortions, and severe class imbalance.

To address the challenges of data imbalance, limited feature integration, and suboptimal parameter tuning in automated breast cancer diagnosis, this study introduces DEViTNeXt, a new hybrid deep learning framework designed to enhance mammogram classification. The framework employs three core strategies: (i) hybrid CNN-Transformer feature extraction to capture both local and global representations, (ii) DE-based optimization of hyperparameters (e.g., learning rate, fusion coefficient) for enhanced stability and adaptability, and (iii) advanced augmentation combining GAN-based synthesis with geometric transformations to mitigate class imbalance and improve data diversity. This design ensures superior classification accuracy, model generalization, and training stability under constrained medical imaging conditions. The following section outlines the specific motivations behind this work and details the key contributions that distinguish our proposed approach from existing methods.

### Motivation and contributions

Breast cancer is a leading cause of mortality in women globally, with early detection being critical for enhancing survival outcomes. Despite remarkable progress in deep learning–based diagnostic systems, several challenges often hinder the accuracy and generalization of such models. First, mammographic datasets are typically small and exhibit strong class imbalance, particularly with underrepresented malignant lesions. Second, CNNs, though effective at capturing local textures, often fail to model long-range dependencies essential for identifying subtle, distributed malignancies. Third, while capturing global context, pure Transformer-based models require large-scale training data and are prone to overfitting on limited medical datasets. These limitations motivate the development of a hybrid framework that jointly exploits local and global feature representations while maintaining robustness and computational efficiency.

To tackle these challenges, this study introduces DEViTNeXt, a new hybrid deep learning model that combines the strengths of ConvNeXt and ViT for mammographic breast cancer classification. The ConvNeXt branch efficiently captures fine-grained local details, whereas the ViT branch models global contextual dependencies across breast tissue regions. A Multi-Head Attention (MHA) mechanism is employed to refine feature representations from both branches. The complementary features are combined through a custom fusion layer, designed to optimize the integration of local and global representations. Furthermore, to enhance the model’s generalization and performance stability, the DE algorithm is used to optimize hyperparameters, automatically tuning learning rate, weight decay, dropout rate, attention head dimension, and fusion weight coefficient. Furthermore, to mitigate dataset imbalance and improve data diversity, a hybrid augmentation strategy is adopted, combining Generative Adversarial Networks (GANs) for synthetic sample generation with geometric transformations to further increase variability. This integrated approach enables the model to learn from a more balanced and representative dataset, thereby improving classification performance across both benign and malignant categories.

The major contributions of this study are summarized as follows:

- A new hybrid architecture (DEViTNeXt) was proposed, which is a unified framework that fuses ConvNeXt’s convolutional efficiency with ViT’s attention-based representation learning, enabling balanced extraction of both local and global features from mammograms.
- A Multi-Head Attention mechanism is incorporated in both feature extraction branches to enhance feature representation and focus on diagnostically significant regions.
- A custom fusion layer is designed to optimally integrate global and local feature maps, improving discriminative power and model interpretability.
- The DE algorithm is used to automatically tune key hyperparameters, including learning rate, weight decay, dropout rate, attention head dimension, and fusion weight coefficient, achieving optimal performance without manual intervention.
- A hybrid augmentation pipeline combining GAN-based synthesis and geometric transformations is developed to mitigate class imbalance and enhance dataset diversity, thereby improving generalization.
- Extensive experiments on two benchmark datasets (CBIS-DDSM and MIAS) demonstrate that DEViTNeXt surpasses several state-of-the-art models in terms of classification accuracy, sensitivity, and robustness.

To delineate the methodological novelty of DEViTNeXt, several distinctive aspects are emphasized in comparison to existing hybrid CNN-Transformer architectures. Although hybrid CNN-Transformer architectures have been explored in recent literature, DEViTNeXt introduces several targeted methodological advancements specifically designed to address the unique challenges of mammographic breast cancer classification. Unlike most existing hybrids that rely on simple feature concatenation or late fusion, our framework implements (i) independent Multi-Head Attention (MHA) refinement modules applied directly to both the ConvNeXt and ViT branches prior to fusion. This enables each stream to dynamically focus on diagnostically critical patterns (fine-grained textures in ConvNeXt and long-range contextual relationships in ViT) before integration. (ii) A DE-optimized MHA-based cross-attention fusion layer that adaptively learns the optimal integration of complementary features, including the fusion coefficient *α*, which is jointly optimized with other critical hyperparameters. (iii) An end-to-end optimization-aware pipeline where Differential Evolution simultaneously tunes architectural and training parameters while being tightly coupled with the hybrid augmentation and composite loss function. These proposed techniques overcome the limitations of manually tuned fusion strategies and the suboptimal feature integration commonly observed in prior hybrid models.

### Paper structure

This paper is structured as follows: Section Literature review reviews the existing literature, Section Methodology outlines the proposed methodology for breast cancer classification, Section Results and discussion presents and discusses the experimental results, and Section Conclusion and future work provides a summary of the conclusions.

## Literature review

Recent advancements in DL and computer vision have transformed medical image analysis, particularly for breast cancer detection, by enabling CAD systems that enhance diagnostic accuracy and support radiologists^40–44^. This section reviews key research efforts in breast cancer classification, focusing on machine learning enhanced DL approaches, CNNs, transformer-based models, and hybrid CNN-Transformer architectures, highlighting their contributions and limitations in mammography and related modalities.

### ML-enhanced deep learning approaches

Machine learning-enhanced DL models offer interpretable and computationally efficient alternatives by integrating traditional ML with neural networks^2,45–47^. Wani et al.^45^ proposed the “BC” model, combining CNNs with Light Gradient Boosting (LGBM) and SHAP for explainability, achieving 98.29% accuracy on a real-world dataset, though single-dataset validation limits broader applicability. Nissar et al.^2^ introduced MOB-CBAM, a lightweight MobileNet-V3 with CBAM, achieving 99% accuracy on CMMD, 98% on CBIS-DDSM, and 97% on MIAS, but cross-modality generalizability requires further study. Song et al.^46^ used GLCM and HOG features with SVM and XGBoost on DDSM, with XGBoost achieving 84% accuracy, demonstrating ensemble methods’ potential. Malebary et al.^47^ integrated k-means, LSTM, CNN, and Random Forest on MIAS and DDSM, achieving 95%–96% accuracy, but optimization for real-time deployment remains a challenge. These approaches underscore the value of ML and DL fusion for interpretability and efficiency but highlight the need for cross-dataset validation and optimization for clinical use.

### CNN-based approaches

Convolutional neural networks have become a cornerstone of breast cancer classification due to their robust feature extraction capabilities for mammography and ultrasound datasets^48–57^. Ting et al.^48^ proposed a customized CNN with manual parameter tuning and augmentation (rotations, flips) on the MIAS dataset, achieving 90.50% accuracy but limited by scalability due to manual optimization. Maqsood et al.^55^ developed a transferable texture CNN (TTCNN) with CLAHE preprocessing, attaining 99.08% accuracy, 99.19% sensitivity, and 98.96% specificity on the DDSM dataset, though its generalizability across modalities requires further exploration. Similarly, Muduli et al.^53^ utilized a deep CNN with four convolutional layers on DDSM, but its high computational cost and limited feature diversity highlight the need for optimization. Rahman et al.^54^ employed a pre-trained ResNet-50 on INbreast, achieving 93% accuracy, but its performance on small datasets limits early-stage cancer detection.

Advanced CNN architectures have also incorporated segmentation and multi-view analysis. Singh et al.^56^ used a conditional GAN (cGAN) for mammogram segmentation on DDSM, followed by shape descriptor-based CNN classification, though its reliance on specific descriptors limits adaptability. Khan et al.^50^ introduced a multi-view feature fusion (MVFF) CAD system with parallel CNNs on CBIS-DDSM and mini-MIAS, achieving 93.73% accuracy by integrating multiple mammographic views. Raaj et al.^52^ proposed a hybrid CNN with morphological segmentation, reporting 98.44% accuracy on DDSM and 98.04% on MIAS, but class imbalance remains a challenge. Atrey et al.^49^ combined CNN with LSTM for sequential modeling, achieving 98.84% accuracy on mammography and 97.16% on ultrasound datasets, demonstrating the value of temporal dependencies. Sahu et al.^57^ developed five hybrid CNN frameworks, with their ShuffleNet-ResNet scheme achieving 99.17% accuracy on mini-DDSM and 96.52% on BUSI, though cross-modality validation is needed. Mahmood et al.^51^ fine-tuned a deep ConvNet with SVM classification on MIAS, reaching 97.8% accuracy, but extensive augmentation was required to mitigate overfitting. These studies highlight CNNs’ strengths in local feature extraction while underscoring limitations in handling class imbalance and long-range dependencies.

Recent studies have made significant contributions to improving synthetic mammogram generation and hybrid modeling. Shah et al.^58^ focused on enhancing the quality and authenticity of GAN-generated mammograms, highlighting the persistent gap between synthetic and real images as perceived by radiologists. Also, the study in^59^ proposed a DCGAN-based synthesis approach to address data scarcity in breast cancer diagnosis. Furthermore, Shah et al.^60^ introduced a dual-view deep learning model to improve detection accuracy by analyzing craniocaudal (CC) and mediolateral oblique (MLO) views. Another ensemble approach by Shah et al.^61^ combined EfficientNet, AlexNet, ResNet, and DenseNet to optimize breast cancer detection.

### Transformer-based approaches

Transformer-based models, leveraging self-attention to capture long-range dependencies, have shown promise in breast cancer classification, particularly for histopathology and mammography^62–66^. Naas et al.^62^ introduced an explainable ViT framework for histopathology images (BreakHis), achieving superior accuracy in binary lesion classification compared to CNNs, though its histopathology focus limits mammography applicability. Similarly, Jahan et al.^63^ developed a three-stage ViT system for whole slide images, attaining 96.74% patch-level accuracy and 89.78% subtype classification accuracy, but cross-modality generalizability remains unaddressed. Mehta et al.^65^ proposed HATNet, a transformer-based algorithm for histopathology, achieving an 8% performance improvement over prior methods with interpretable attention maps, yet its focus on whole slide images restricts its mammography utility. Abimouloud et al.^66^ compared three transformer variants (ViT, CCT, Token Learner) for histopathology patch classification, with CCT achieving 99.92% accuracy and Token Learner offering computational efficiency (534 seconds training). However, their histopathology focus necessitates further mammography validation. In mammography, Manigrasso et al.^64^ developed transformer and graph-based models for multi-view analysis, outperforming CNNs by leveraging relational modeling, but low cancer prevalence and high-resolution processing challenges limit accuracy. These studies demonstrate transformers’ ability to model global context but highlight the need for large datasets and cross-modality validation.

### Hybrid CNN-transformer models

Hybrid CNN-Transformer models combine local feature extraction with global context modeling to address the limitations of standalone architectures^67–71^. Zeynali et al.^67^ proposed a CNN-Transformer framework using Xception and a transformer on BreakHis and IDC datasets, achieving 96.15%–99.62% accuracy, though restricted to histopathology. Hayat et al.^68^ introduced an EffNetV2–ViT hybrid, attaining 99.83% accuracy for binary classification and 98.10% for eight-class classification on BreakHis, but mammography applications remain unexplored. Boudouh et al.^69^ developed a dual-branch hybrid for mammography (CBIS-DDSM), combining ViT++ and CNNs (Xception, VGG16, RegNetX002), achieving 99.22% accuracy, significantly outperforming standalone VGG16 (61.96%). Furthermore, Huang et al.^70^ proposed a SqueezeNet-ICBO hybrid with noise reduction and feature selection, enhancing efficiency on MIAS, though single-dataset evaluation limits generalizability. Mahmood et al.^71^ combined radiomics with VGGNet and SE-ResNet152, using Grad-CAM for interpretability, achieving near-perfect sensitivity (AUC 0.99), but broader validation is needed. These hybrid approaches highlight the synergy of CNNs and transformers but call for multi-dataset and mammography-focused validation to ensure clinical applicability.

Recent studies have explored various advanced techniques for breast cancer detection across different imaging modalities. Nissar et al.^72^ proposed a comprehensive framework using radiomics features optimized by Differential Evolution and Grey Wolf Optimization for molecular subtype prediction on mammograms. In another work, Nissar et al.^73^ introduced a computationally efficient LC-SCS deep learning model for breast cancer classification using thermal images. Furthermore, Nissar et al.^74^ developed SwinEff-AttentionNet, a hybrid model combining Swin Transformer and EfficientNet with local self-attention for breast ultrasound segmentation and classification. Additionally, Nissar et al.^75^ presented Mod-ViT, a modified Vision Transformer framework evaluated on multiple imaging modalities, including mammograms and ultrasound.

### Research gaps and critical synthesis

Although the literature demonstrates considerable progress in automated breast cancer classification from mammograms, several critical gaps remain, particularly in dynamic feature integration, automated hyperparameter optimization, effective handling of class imbalance, and rigorous patient-level validation across multiple datasets. Table 1 presents a critical comparison of representative state-of-the-art studies, highlighting architectural design, optimization strategy, fusion mechanism, and key limitations.

**Table 1:** Comparison of recent breast cancer classification methods on mammography datasets.

**Study**	**Architecture**	**Optimization**	**Fusion strategy**	**Key limitation**	**Best accuracy (%)**
Ting et al.^48^	Custom CNN	Manual	–	Manual tuning and limited scalability	90.50 (MIAS)
Maqsood et al.^55^	TTCNN	Manual	–	Limited cross-dataset generalization	99.08 (DDSM)
Raaj et al.^52^	Hybrid CNN	Manual	Morphological segmentation	Class imbalance challenge	98.44 (DDSM)
Khan et al.^50^	Multi-view CNN	Manual	Feature fusion	Dependency on multi-view imaging	93.73 (CBIS-DDSM)
Muduli et al.^53^	Deep CNN	Manual	–	High computational complexity	High on DDSM
Naas et al.^62^	ViT	Manual	Self-Attention	Histopathology-focused and data hungry	High on BreakHis
Jahan et al.^63^	Three-stage ViT	Manual	Self-Attention	Limited mammography validation	96.74 (patch-level)
Boudouh et al.^69^	ViT++ + CNNs	Manual	Late fusion	Static fusion mechanism	99.22 (CBIS-DDSM)
Hayat et al.^68^	EfficientNetV2 + ViT	Manual	Concatenation	Primarily validated on histopathology	99.83 (BreakHis)
Zeynali et al.^67^	Xception + Transformer	Manual	Concatenation	Histopathology-oriented evaluation	99.62 (BreakHis)
**Proposed DEViTNeXt**	**ConvNeXt + ViT**	**Differential Evolution**	**DE-optimized MHA Cross-Attention**	**Designed for robust mammography classification with optimized feature fusion**	**99.58 (CBIS-DDSM)**

As illustrated in Table 1, CNN-based models generally provide strong local feature extraction but suffer from manual hyperparameter tuning and limited ability to model long-range dependencies. Transformer-based approaches effectively capture global context yet are often data-intensive and less validated on mammography datasets. Most hybrid CNN-Transformer models still rely on static fusion strategies (concatenation or late fusion) and manual optimization. The proposed DEViTNeXt addresses these gaps through independent Multi-Head Attention refinement on both branches, a Differential Evolution-optimized dynamic cross-attention fusion layer, and a hybrid GAN-geometric augmentation pipeline applied strictly after patient-level splitting.

## Methodology

**Fig. 1:**
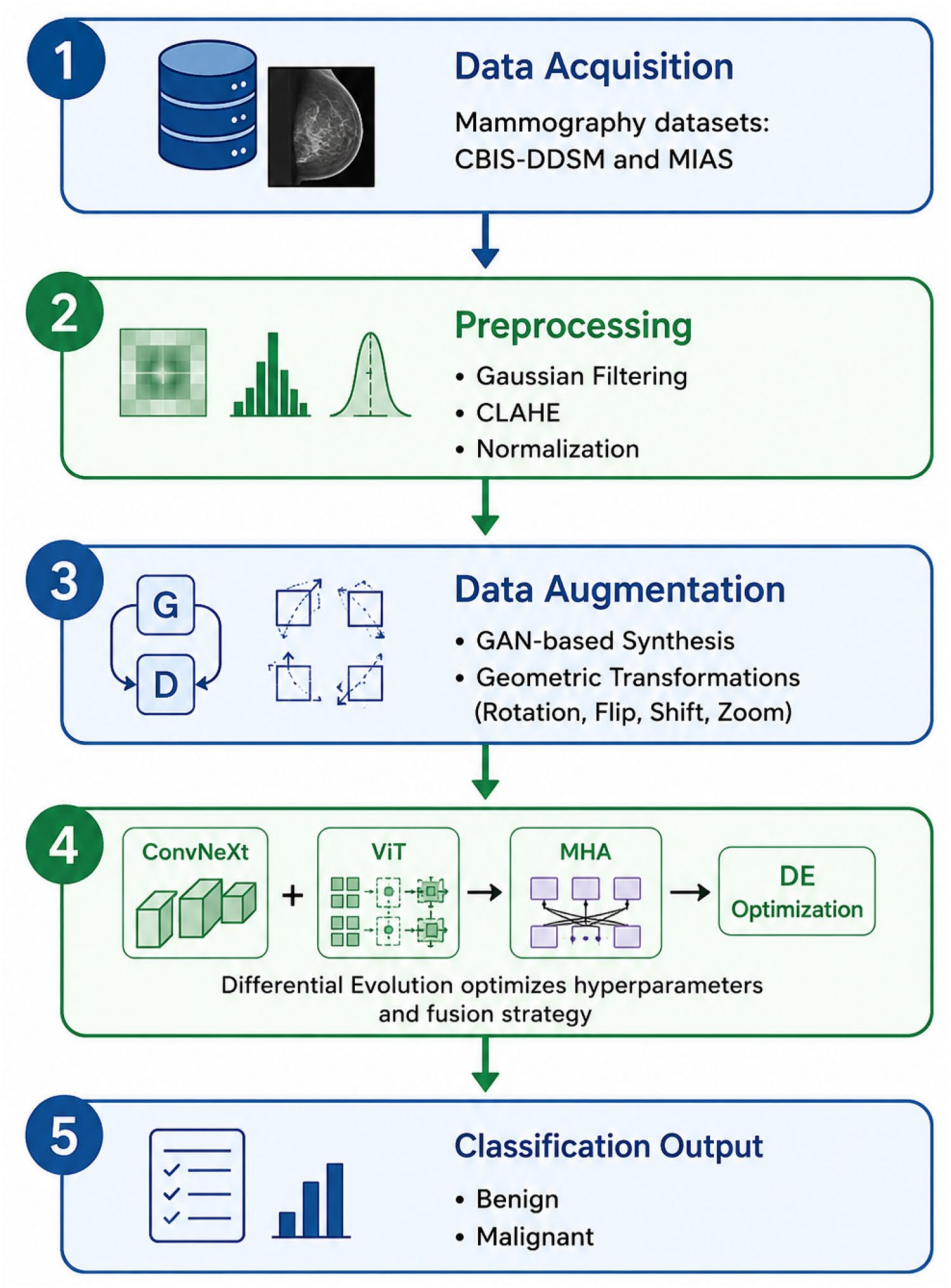
Overview of the proposed DEViTNeXt framework.

This paper introduces DEViTNeXt, a novel hybrid deep learning framework that integrates ConvNeXt convolutional networks, ViT, and DE optimization for automated breast cancer classification from mammographic images. These design choices are informed by the unique characteristics of mammograms, which exhibit high local textural variability, such as microcalcifications and mass margins, alongside a requirement for global contextual understanding, including tissue asymmetry and architectural distortion. Conventional CNNs are limited in capturing long-range dependencies, whereas standalone ViTs lack the inductive biases necessary for fine-grained medical textures. .Figure 1 illustrates the complete DEViTNeXt architecture, presenting the end-to-end pipeline from data preprocessing through feature extraction, attention-based fusion, and optimized classification. The framework begins with comprehensive preprocessing of the CBIS-DDSM and MIAS datasets, including Gaussian noise removal, Contrast Limited Adaptive Histogram Equalization (CLAHE), and min–max intensity normalization to standardize mammographic inputs while preserving diagnostically critical features. To address class imbalance and limited sample sizes, a hybrid augmentation strategy combines GAN-based synthetic mammogram generation with geometric transformations.

At the core of DEViTNeXt lies a dual-branch feature-extraction mechanism that leverages ConvNeXt for hierarchical local feature extraction and ViT for global contextual modeling via patch-based self-attention. Each branch integrates Multi-Head Attention (MHA) refinement to emphasize diagnostically salient regions–ConvNeXt MHA captures long-range spatial dependencies, while ViT MHA enhances semantic consistency (Section 3.4.3). An MHA-based feature fusion module (Section Feature fusion using multi-head attention (MHA)) adaptively integrates the complementary ConvNeXt and ViT representations via cross-attention, balancing local textural precision with global tissue context. Key hyperparameters–including learning rate (*η*), weight decay (*λ*), dropout (*p*), attention heads ($d_h$), and fusion coefficient (*α*)–are optimized using Differential Evolution (DE) (Section Differential evolution for hyperparameter optimization), a population-based evolutionary algorithm that surpasses PSO, GWO, and Grid Search through adaptive mutation and crossover operators.

Finally, a composite loss function combining Weighted Cross-Entropy and Focal Loss (Section Implementation of composite loss function) mitigates class imbalance while emphasizing challenging malignant cases, ensuring stable and robust convergence. The complete DEViTNeXt pipeline achieves state-of-the-art performance by synergistically combining preprocessing robustness, architectural complementarity, attention-driven refinement, evolutionary optimization, and imbalance-aware training–positioning it as a clinically viable solution for automated breast cancer screening.

### Dataset description

This study evaluates the proposed model’s performance using two public benchmark datasets, the Curated Breast Imaging Subset of DDSM (CBIS-DDSM) and the Mammographic Image Analysis Society (MIAS) database. Mammography, vital for early breast cancer detection, identifies masses and calcifications in X-ray images^76^. To guarantee that no patient appears in more than one split, the data partitioning was performed at the patient level rather than at the individual image level using the supplied patient IDs. This ensures that the model is evaluated on completely unseen patients, providing a more realistic assessment of its generalization capability for clinical deployment.

#### CBIS-DDSM dataset

The CBIS-DDSM is a standardized, updated version of the Digital Database for Screening Mammography (DDSM)^77^. This curated dataset offers significant improvements, including images in the standardized DICOM format and a restructured, more accessible metadata organization. It comprises 1,459 mammograms annotated across four diagnostic categories: Benign Calcification (398 images), Benign Mass (417 images), Malignant Calcification (300 images), and Malignant Mass (344 images). All mammograms were preprocessed by resizing them to a uniform resolution of *224 × 224* pixels. We formulated the problem as a binary classification task (Benign vs. Malignant) by grouping Benign Calcification and Benign Mass into one class (“Benign”) and Malignant Calcification and Malignant Mass into another class (“Malignant”). This mapping is consistent with common clinical practice in breast cancer screening, where the primary goal is to distinguish malignant lesions requiring immediate intervention from benign ones.

#### MIAS dataset

The MIAS database was used to assess the robustness and generalizability of the proposed model^78^. This dataset, assembled by a collection of UK research groups, includes 322 mammograms divided into three classes: benign (63), malignant (52), and normal (207). A summary of the key characteristics of both datasets is provided in Table 2.

**Table 2:** Summary of the datasets used in this study.

**Attribute**	**CBIS-DDSM**	**MIAS**
Total number of images	1,459	322
Image dimension	*224 × 224*	*224 × 224*
Image type	Digital mammogram	Digital mammogram
*Class distribution*		
Benign calcification	398	–
Benign mass	417	–
Malignant calcification	300	–
Malignant mass	344	–
Benign	–	63
Malignant	–	52
Normal	–	207
Availability	Public	Public

### Data preprocessing

#### Noise removal

A Gaussian filter is applied to each image. The Gaussian filter smooths an image by convolving it with a Gaussian function, effectively suppressing high-frequency noise while preserving edge integrity. Mathematically, the Gaussian function is defined by Eq. (1):

1 $$\begin{aligned} G(a, b) = \frac{1}{2\pi \sigma ^2} \exp \left( -\frac{a^2 + b^2}{2\sigma ^2}\right) \end{aligned}$$

where *G*(*a*, *b*) represents the Gaussian filter value at spatial coordinates (*a*, *b*), and *σ* denotes the standard deviation, which controls the degree of smoothing.

In this study, a kernel size of *5 × 5* with *σ = 1.0* is utilized, providing an optimal balance between noise reduction and preservation of fine anatomical details.

#### Intensity normalization and resizing

The initial preprocessing stage transforms the original mammogram intensity values to a standardized range. We use min-max normalization to rescale pixel intensities to [0, 1], as shown in Eq. (2)^5^:

2 $$\begin{aligned} \textbf{I}_{\text {norm}} = \frac{\textbf{I} - I_{\text {min}}}{I_{\text {max}} - I_{\text {min}}} \end{aligned}$$

where $\textbf{I}$ represents the original input mammogram with minimum and maximum intensity values $I_{\text {min}}$ and $I_{\text {max}}$ respectively, and $\textbf{I}_{\text {norm}}$ denotes the normalized output image.

Following intensity normalization, all mammograms were resized to *224 × 224* pixels to balance computational efficiency, memory constraints on standard hardware, and compatibility with pre-trained ConvNeXt and ViT backbones. While aggressive downsampling may lead to some loss of fine-grained details such as small microcalcifications, preliminary experiments with higher resolutions (e.g., *512 × 512*) showed only marginal gains in performance (*<0.8%*) at the cost of more than *3×* increase in training time and GPU memory, making *224 × 224* a practical and effective choice for the proposed framework.

#### Image enhancement using CLAHE

To enhance the visibility of subtle breast tissue structures and improve diagnostic interpretability, Contrast-Limited Adaptive Histogram Equalization (CLAHE) is employed as a local contrast enhancement method. The algorithm partitions the mammogram into a grid of non-overlapping tiles, computes each tile’s pixel intensity histogram, and clips it using a predefined limit to avoid noise amplification. The clipped pixels are then evenly redistributed across intensity levels, achieving balanced local contrast enhancement without artifacts^51^.

The total number of tiles, denoted by *T*, is defined as (3):

3 $$\begin{aligned} T = \frac{I_x}{t_x} \times \frac{I_y}{t_y} \end{aligned}$$

where $I_x$ and $I_y$ represent the image dimensions along the horizontal and vertical axes, respectively, and $t_x$ and $t_y$ denote the dimensions of each tile.

To further control the enhancement strength, the algorithm utilizes a Normalized Contrast Limit (*NCL*), while the average number of pixels per grayscale level, $N_{\text {avg}}$, is calculated by Eq. (4):

4 $$\begin{aligned} N_{\text {avg}} = \frac{I_x \times I_y}{G} \end{aligned}$$

where *G* is the total number of grayscale intensity levels.

The mean number of clipped pixels per bin, $N_{\text {clip}}$, is determined by Eq. (5):

5 $$\begin{aligned} N_{\text {clip}} = \frac{\sum C_L}{G} \end{aligned}$$

where $\sum C_L$ denotes the total number of pixels exceeding the defined clip limit.

Any remaining pixels that are not distributed through clipping are uniformly spread across all intensity bins according to Eq. (6):

6 $$\begin{aligned} R = \frac{G}{N_r} \end{aligned}$$

where $N_r$ represents the number of remaining unallocated pixels.

### Data augmentation

Medical imaging datasets for breast cancer detection are often limited in size and suffer from class imbalance, which can negatively affect the performance and generalization ability of deep learning models. In particular, the MIAS dataset contains a relatively small number of samples, while the CBIS-DDSM dataset exhibits an uneven distribution of benign and malignant cases, with malignant lesions being underrepresented. These issues increase the risk of overfitting and hinder the model’s ability to learn robust diagnostic features. To address these challenges, this study employs a hybrid augmentation strategy that integrates Generative Adversarial Networks (GANs)^79^ with geometric transformation techniques. The GAN-based augmentation was used to generate realistic mammogram samples, particularly for the minority malignant class, thereby alleviating class imbalance. In parallel, geometric transformations, such as rotation, flipping, scaling, and translation, were applied to enhance spatial variability and simulate real-world imaging conditions.

The overall distribution of the datasets before and after augmentation is summarized in Table 3. All data augmentation, including GAN-based synthesis of malignant cases, was performed only after splitting the data at the patient level and only on the training set of each fold. This kept the test set free from synthetic samples, allowing for an unbiased evaluation of how well the model generalizes. The MIAS dataset was augmented from 322 to 1,050 images to achieve better class balance. The target class size (approximately 340–360 samples) was chosen to provide sufficient training data for the minority malignant class while avoiding excessive synthetic samples that could introduce distribution shift. This augmentation ratio was determined through preliminary experiments to optimize the trade-off between data diversity and training stability.

#### GAN-based data augmentation

To address severe class imbalance and the limited number of annotated mammograms, a conditional deep convolutional GAN (cDCGAN) was employed for synthetic data generation. This was applied exclusively on the training set after patient-level splitting to prevent data leakage.

**Fig. 2:**
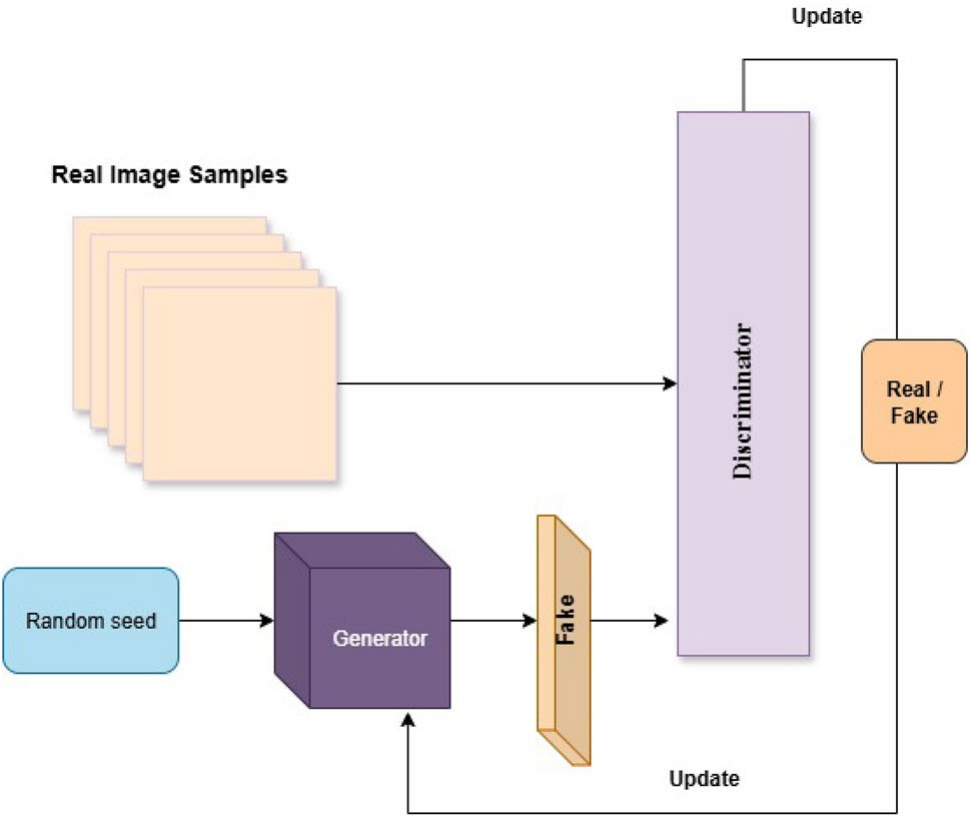
Architecture of the conditional Deep Convolutional GAN (cDCGAN) used for mammogram synthesis.

The overall GAN architecture is illustrated in Figure 2. The generator consists of five transposed convolutional layers with batch normalization and ReLU activations, while the discriminator comprises five convolutional layers with LeakyReLU activations (negative slope 0.2).

Training was conducted for 250 epochs on CBIS-DDSM and 350 epochs on MIAS using the Adam optimizer with a learning rate of $2\times 10^{-4}$, $\beta _1 = 0.5$, and $\beta _2 = 0.999$, and a mini-batch size of 16. The objective combined a non-saturating adversarial loss with an L1 reconstruction loss (*λ =100*) to preserve fine anatomical structures. Early stopping was applied when the discriminator accuracy stabilized around 50% for 10 consecutive epochs.

**Fig. 3:**
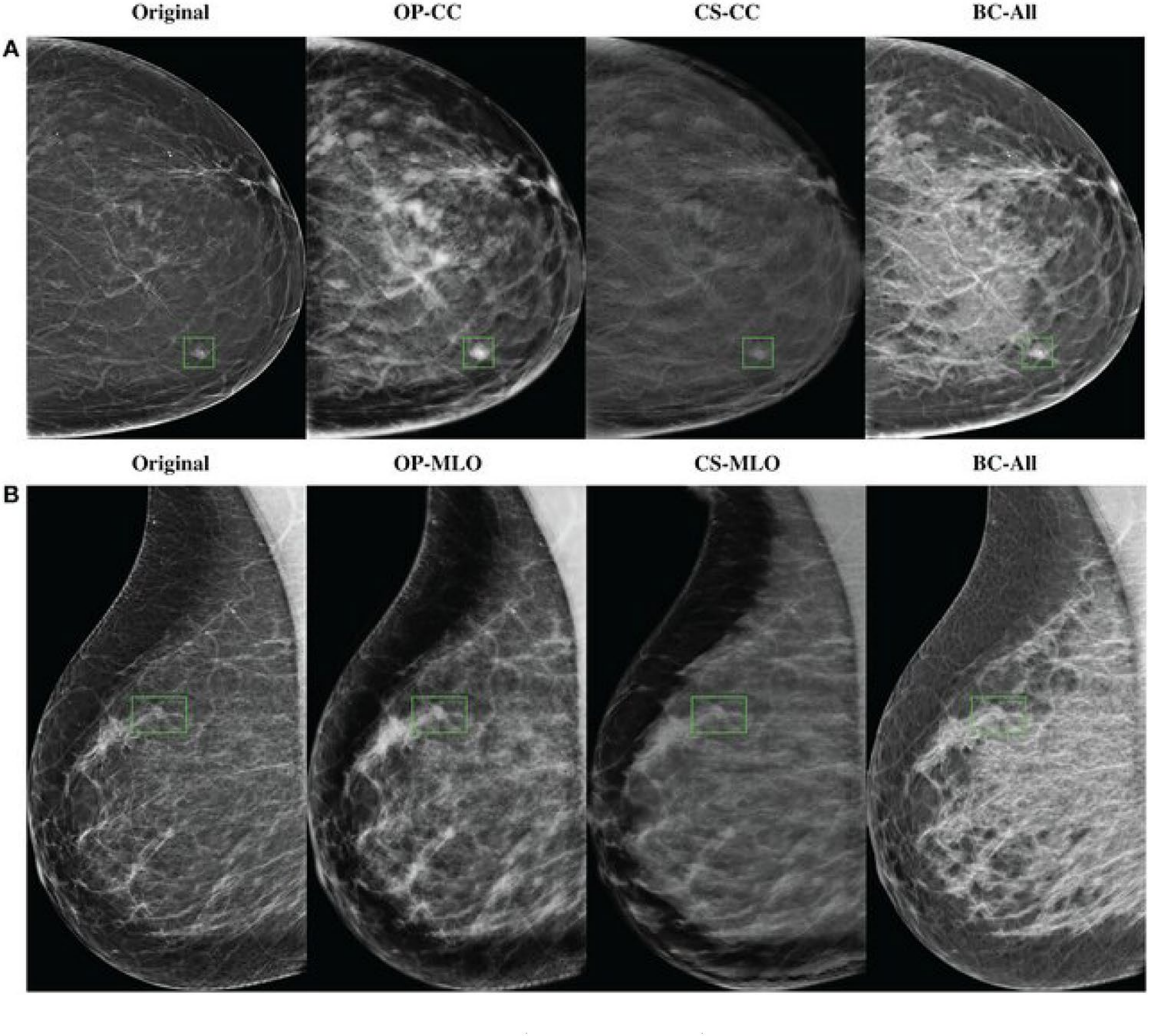
Representative examples of real mammograms (left column) and GAN-generated synthetic mammograms (right columns). Green boxes highlight preserved critical diagnostic features.

The quality of the generated mammograms was evaluated both quantitatively and qualitatively. Quantitatively, the Fréchet Inception Distance (FID) score was 17.8, indicating high visual fidelity and close distribution matching with real mammograms. Additionally, Structural Similarity Index (SSIM) and Peak Signal-to-Noise Ratio (PSNR) were computed between the synthetic and real images, yielding an average SSIM of 0.68 and PSNR of 24.7 dB. These values confirm that the generated samples maintain realistic appearance while being sufficiently different from the original training images, thereby avoiding direct copying and potential data leakage. Qualitative visual inspection (Figure 3) further verified the preservation of key diagnostic features such as microcalcifications, mass margins, and tissue density patterns without noticeable artifacts.

The adversarial training objective is given by:

7 $$\begin{aligned} \mathcal {L}_{\text {GAN}}(\mathcal {G}, \mathcal {D}) = \min _{\mathcal {G}} \max _{\mathcal {D}} \left\{ \mathbb {E}_{\textbf{x} \sim p_{\text {data}}(\textbf{x})}[\log \mathcal {D}(\textbf{x})] + \mathbb {E}_{\textbf{z} \sim p_{\textbf{z}}(\textbf{z})}[\log (1 - \mathcal {D}(\mathcal {G}(\textbf{z})))] \right\} . \end{aligned}$$

Our implementation followed dataset-specific strategies:

- **MIAS Dataset:** GANs generated substantial additional samples, particularly for the malignant class, to overcome the small dataset size.
- **CBIS-DDSM Dataset:** GANs were selectively used to synthesize malignant cases and mitigate class imbalance.

#### Geometric transformations

In addition to GAN synthesis, standard geometric augmentation techniques were employed to enhance the robustness of the hybrid ConvNeXt–ViT model. Each mammogram in the training set was randomly subjected to one or more of the following transformations:

- **Rotation:** random rotations within $\pm 15^\circ$ to simulate variations in breast positioning.
- **Flipping:** horizontal and vertical flips to account for anatomical symmetry between left and right breast views.
- **Scaling:** resizing by a factor of 0.9–1.1 to accommodate variations in breast size and imaging magnification.
- **Translation:** shifting along the x and y axes by up to 10% of the image size to mimic positional changes during acquisition.

**Table 3:** Final dataset distribution after GAN- and geometric-based augmentation.

**Class category**	**CBIS-DDSM (original)**	**CBIS-DDSM (augmented)**	**MIAS (original)**	**MIAS (augmented)**
**Total number of images**	1,459	5,950	322	1,050
*Detailed class distribution*				
Benign calcification	398	1,360	–	–
Benign mass	417	1,445	–	–
Malignant calcification	300	1,400	–	–
Malignant mass	344	1,445	–	–
Benign	–	–	63	350
Malignant	–	–	52	340
Normal	–	–	207	360

### ConvNeXt and ViT feature extraction

The architectural design of DEViTNeXt is driven by the complementary limitations observed in prior medical imaging literature. ConvNeXt provides strong hierarchical local feature extraction with modernized convolutional inductive biases, while ViT excels at modeling global dependencies. The independent MHA refinement on each branch, followed by a DE-optimized MHA-based cross-attention fusion strategy, was deliberately chosen to maximize feature complementarity rather than relying on simple concatenation or late fusion, as is common in many existing hybrid models. This design is empirically validated through comprehensive ablation studies (Section Ablation studies).

This complements ConvNeXt’s localized feature extraction, providing a comprehensive representation of mammographic content. MHA is applied independently to both ConvNeXt and ViT outputs, refining representations by dynamically weighting diagnostically significant regions. This attention mechanism enhances discriminative power, ensuring that local and global features contribute effectively to accurate diagnosis.

Both ConvNeXt and ViT backbones were initialized with weights pre-trained on ImageNet, a common and effective practice in medical imaging when dealing with limited annotated data. This transfer learning strategy allows the models to leverage rich low-level and mid-level features learned from natural images. During training, we performed end-to-end fine-tuning of all layers with a lower learning rate for the backbone (10% of the classifier learning rate) to adapt the pre-trained features to the characteristics of mammographic images while preventing catastrophic forgetting.

**Fig. 4:**
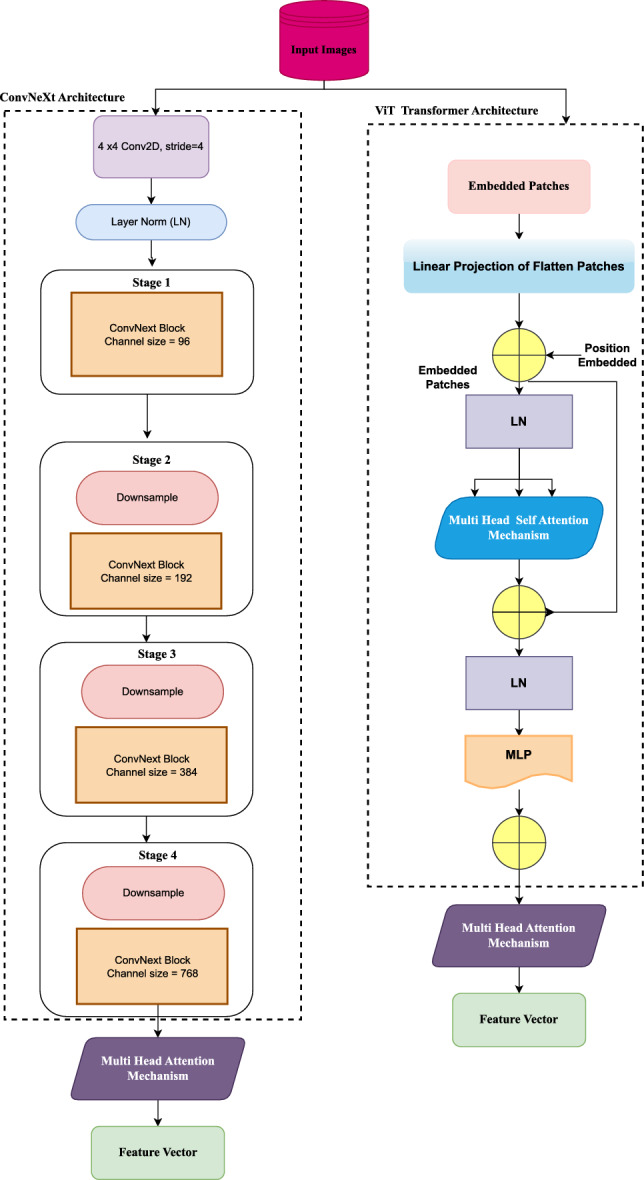
Feature extraction model.

Figure 4 depicts the feature extraction pipeline, showing how ConvNeXt, ViT, and MHA collaboratively generate robust, context-aware, and clinically relevant feature representations for precise breast cancer classification.

#### ConvNeXt block for feature extraction

The ConvNeXt architecture^80^ represents a modernized multilayer convolutional framework designed for advanced image processing tasks, including mammography analysis. Originally developed to compete with state-of-the-art vision transformers such as Swin Transformers^81^, ConvNeXt incorporates several architectural advancements from transformers while maintaining the fundamental principles of convolutional networks.

**Fig. 5:**
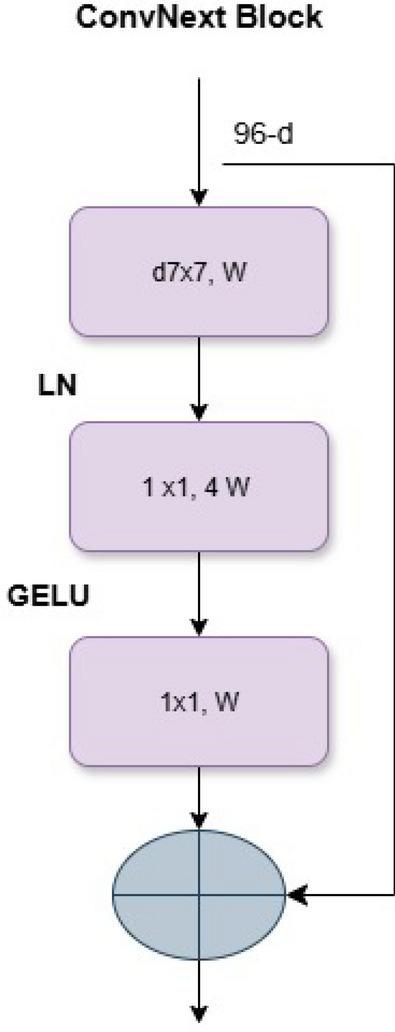
Basic block design of ConvNeXt architecture.

As illustrated in Figure 5, the ConvNeXt block introduces several key innovations that enhance its suitability for medical image analysis^80^. First, it replaces batch normalization with layer normalization^82^ to improve training stability and performance. Layer normalization normalizes inputs across feature dimensions rather than batch dimensions, which is particularly beneficial for mammography datasets that often exhibit varying intensity distributions. The normalization operation is defined by Eq. (8):

8 $$\begin{aligned} \text {LN}(x) = \gamma \cdot \frac{x - \mu }{\sqrt{\sigma ^2 + \epsilon }} + \beta \end{aligned}$$

where *μ* and *σ* represent the mean and standard deviation of input *x*, *γ* and *β* are learnable scaling and shifting parameters, and *ε* ensures numerical stability. In ConvNeXt blocks, layer normalization is typically applied after depthwise convolution operations and before activation functions to stabilize training dynamics.

Second, the downsampling mechanism in ConvNeXt employs *2 × 2* convolutional layers with a stride of 2, replacing the traditional approach used in residual networks. This strategy, combined with strategically placed normalization layers, contributes to more stable training and improved accuracy in mammography classification tasks^80^. Third, ConvNeXt simplifies the conventional convolutional design by reducing the number of activation and normalization layers while implementing an inverted bottleneck structure. This design expands the hidden dimension of the multi-layer perceptron block to four times the input dimension, drawing inspiration from MobileNetV2 to enhance computational efficiency and model performance for medical imaging applications.

The activation function employed in ConvNeXt is the Gaussian Error Linear Unit (GELU)^83^, which introduces non-linearity through a probabilistic transformation. The GELU activation is mathematically defined by Eq. (9):

9 $$\begin{aligned} \text {GELU}(x) = x \Phi (x) \end{aligned}$$

where *x* denotes the input and *Φ (x)* represents the cumulative distribution function of the standard normal distribution. This can be equivalently expressed using Eq. (10):

10 $$\begin{aligned} \text {GELU}(x) = \frac{x}{2} \left[ 1 + \text {erf}\left( \frac{x}{\sqrt{2}} \right) \right] \end{aligned}$$

where $\text {erf}(x)$ is the error function. For practical implementation, the following approximation is commonly used^83^:

11 $$\begin{aligned} \text {GELU}(x) \approx 0.5x \left( 1 + \tanh \left[ \sqrt{\frac{2}{\pi }} \left( x + 0.044715x^3 \right) \right] \right) \end{aligned}$$

#### Vision transformer for feature extraction

**Fig. 6:**
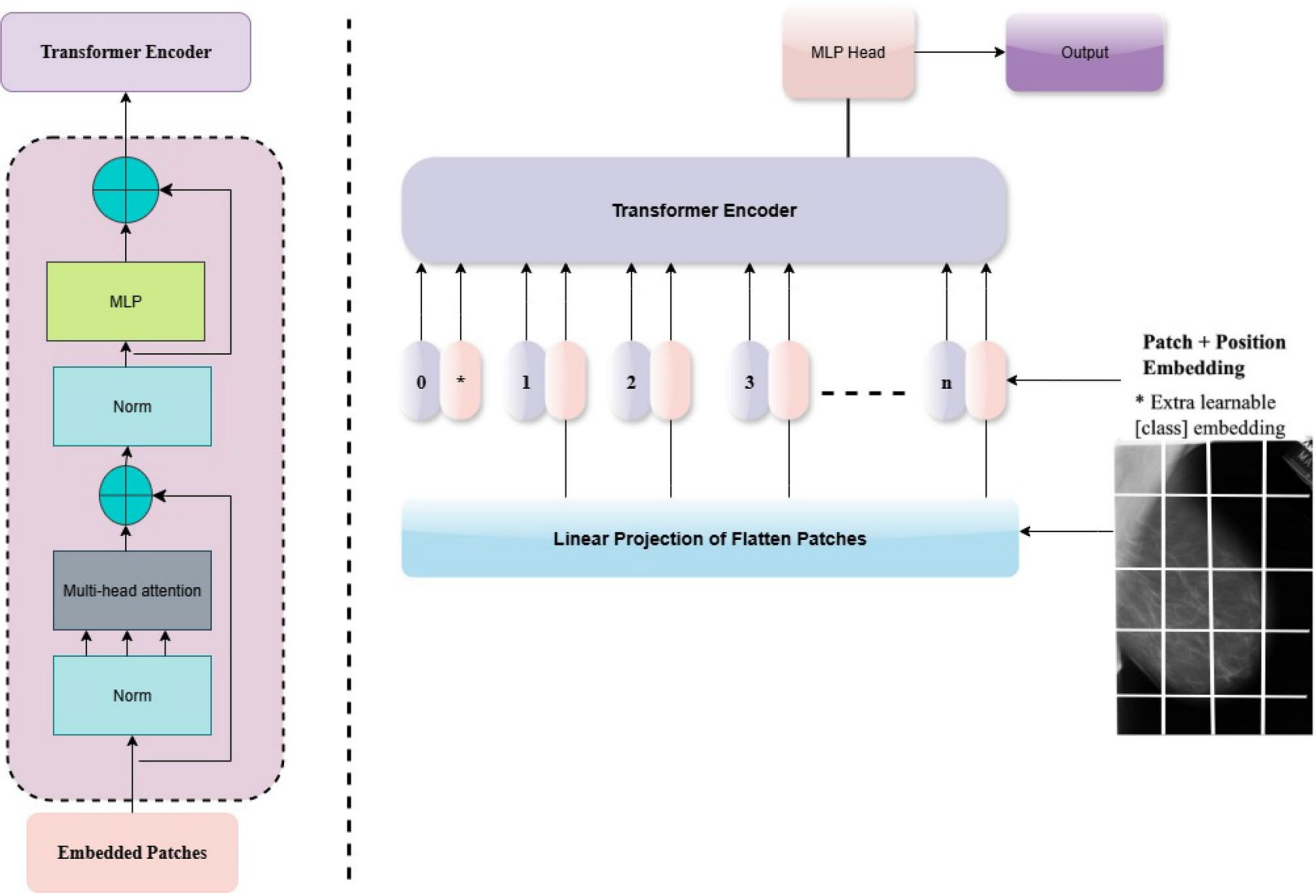
ViT architecture.

The Vision Transformer (ViT)^19^ revolutionizes computer vision by adapting the transformer architecture, originally developed for natural language processing, to image analysis. Unlike CNNs with their inherent inductive biases, ViT segments an image into fixed-size patches, linearly embeds them, and processes them through a standard transformer encoder, as shown in Figure 6. This approach enables ViT to capture global contextual relationships across all patches from the start. ViT’s ability to model complex visual dependencies stems from its multi-head self-attention mechanism. Each encoder block projects the input sequence into query, key, and value vectors, computing a weighted sum of values based on attention scores derived from query-key compatibility.

The Vision Transformer processes input images $\textbf{I} \in \mathbb {R}^{H \times W \times C}$ by first dividing them into *n* patches of size *p × p*, where $n = \frac{H \times W}{p^2}$. These patches are flattened and mapped through a trainable linear projection layer to produce embeddings in a higher-dimensional space suitable for transformer operations. The resulting 2D matrix $\textbf{Z}_{\text {LP}}$ is transformed into 1D embedding vectors $\textbf{Z}$, with each patch embedding calculated using Eq. (12):

12 $$\begin{aligned} \textbf{Z}_i^o = \textbf{x}_i \cdot \textbf{E} + \textbf{E}_{\text {pos}} \end{aligned}$$

where $\textbf{x}_i$ denotes the flattened patch, $\textbf{E}$ represents the learnable projection matrix, and $\textbf{E}_{\text {pos}}$ signifies the positional embedding that encodes spatial information.

To manage computational demands while preserving spatial context, sinusoidal positional encodings $\textbf{P}_\beta$ are utilized. These embeddings alternate between sine and cosine functions based on position and dimension as presented in Eq. (13):

13 $$\begin{aligned} \textbf{P}_{\beta ,i} = {\left\{ \begin{array}{ll} \sin \left( \frac{\beta }{10000^{2i/m}}\right) & \text {for even } i \\ \cos \left( \frac{\beta }{10000^{2i/m}}\right) & \text {for odd } i \end{array}\right. } \end{aligned}$$

In Eq. (13), *β* indicates the position index, *i* the dimension, and *m* the maximum sequence length. The complete patch representation $\boldsymbol{\psi }$ is formed by concatenating the linearly projected patches with their positional encodings as shown in Eq. (14):

14 $$\begin{aligned} \boldsymbol{\psi } = \text {concat}(\textbf{Z}_{\text {LP}}, \textbf{P}_\beta ) \end{aligned}$$

The embedded patches are processed through an encoder with eight identical layers, each comprising layer normalization, multi-head attention (MHA), and a multi-layer perceptron (MLP). The attention mechanism projects input embeddings into query ($\textbf{q}$), key ($\textbf{k}$), and value ($\textbf{v}$) matrices using learnable weights.

15 $$\begin{aligned} & \textbf{q} = \boldsymbol{\psi } \textbf{W}_q \end{aligned}$$

16 $$\begin{aligned} & \textbf{k} = \boldsymbol{\psi } \textbf{W}_k \end{aligned}$$

17 $$\begin{aligned} & \textbf{v} = \boldsymbol{\psi } \textbf{W}_v \end{aligned}$$

The scaled dot-product attention is computed as shown in Eq.(18):

18 $$\begin{aligned} \text {Att}(\textbf{q}, \textbf{k}, \textbf{v}) = \text {softmax}\left( \frac{\textbf{q}\textbf{k}^\top }{\sqrt{D_k}}\right) \textbf{v} \end{aligned}$$

The multi-head mechanism combines outputs from multiple attention heads according to Eq.(19):

19 $$\begin{aligned} \text {MHA}(\textbf{q}, \textbf{k}, \textbf{v}) = \text {Concat}(\text {head}_1, \dots , \text {head}_h)\textbf{W}_O \end{aligned}$$

where each attention head is computed independently as defined in Eq. (20):

20 $$\begin{aligned} \text {head}_i = \text {Att}(\boldsymbol{\psi } \textbf{W}_q^i, \boldsymbol{\psi } \textbf{W}_k^i, \boldsymbol{\psi } \textbf{W}_v^i) \end{aligned}$$

The encoder employs residual connections around each sub-layer, followed by layer normalization. The MLP component uses GELU activation to introduce non-linearity, producing the final feature representation as given in Eq. (21):

21 $$\begin{aligned} \textbf{F}_{\text {ViT}} = \text {GELU}(\text {MLP}(\text {MHA}(\textbf{z}))) \end{aligned}$$

This feature vector $\textbf{F}_{\text {ViT}}$ is subsequently passed through an additional multi-head attention block to further enhance the model’s representational capacity.

#### Multi-head attention mechanism

Following feature extraction, two independent MHA layers are employed to model dependencies across spatial and semantic representations, emphasizing diagnostically relevant regions within mammographic images^84^. The first MHA layer operates on the ConvNeXt feature maps, while the second refines the ViT embeddings. This dual-attention strategy enables the model to focus simultaneously on local textural patterns and global contextual cues, thereby improving both classification accuracy and interpretability in breast cancer analysis.

Each MHA layer consists of multiple attention heads, each of which learns to attend to distinct aspects of the image representation. The attention mechanism computes weighted combinations of input features using query, key, and value projections. These weights dynamically highlight regions with high diagnostic relevance, enabling adaptive refinement of both local and global representations.

The enhanced ConvNeXt features after attention refinement are given by:

22 $$\begin{aligned} \textbf{F}_{\text {att}}^{\text {ConvNeXt}} = \text {MHA}(\textbf{F}_{\text {ConvNeXt}}) \end{aligned}$$

Similarly, the ViT features undergo a self-attention refinement process to enhance semantic consistency and contextual focus:

23 $$\begin{aligned} \textbf{F}_{\text {att}}^{\text {ViT}} = \text {MHA}(\textbf{F}_{\text {ViT}}) \end{aligned}$$

The resulting attention-refined feature maps, $\textbf{F}_{\text {att}}^{\text {ConvNeXt}}$ and $\textbf{F}_{\text {att}}^{\text {ViT}}$, encapsulate spatially and semantically salient information essential for robust breast cancer classification. These refined representations are subsequently passed to the fusion module described in Section Feature fusion using multi-head attention (MHA) for joint decision learning.

### Feature fusion using multi-head attention (MHA)

This MHA-based cross-attention fusion layer, optimized via differential evolution, distinguishes our approach from conventional hybrid models by enabling dynamic, data-driven feature integration rather than static concatenation or simple late fusion commonly used in prior studies. The fusion coefficient and attention weights are jointly optimized with other model hyperparameters, allowing adaptive emphasis on the most complementary local-global interactions.

To effectively integrate the complementary representations learned from both networks, a Multi-Head Attention (MHA)-based fusion layer is employed to combine the enhanced feature maps $\textbf{F}_{\text {att}}^{\text {ConvNeXt}}$ and $\textbf{F}_{\text {att}}^{\text {ViT}}$ extracted from the ConvNeXt and Vision Transformer branches, respectively. This fusion mechanism enables comprehensive learning of mammographic characteristics by establishing deeper inter-feature relationships, crucial for accurate lesion discrimination and diagnostic interpretability.

Before fusion, feature normalization is performed using a linear scaling transformation to constrain feature magnitudes within [0,1], ensuring balanced contribution from both branches. The normalized feature maps are concatenated and fed into the multi-head attention module, formulated as:

24 $$\begin{aligned} \textbf{F}_{\text {fused}} = \text {MHA}([\textbf{F}_{\text {ConvNeXt}} \oplus \textbf{F}_{\text {ViT}}]) \end{aligned}$$

where *⊕* denotes the concatenation operation, and MHA represents the multi-head attention operator that computes cross-attention between the ConvNeXt and ViT feature embeddings.

The resulting fused feature vector $\textbf{F}_{\text {fused}}$ is then passed through a fully connected projection layer followed by a softmax classifier to determine the final diagnostic category (benign or malignant).

### Differential evolution for hyperparameter optimization

To further enhance the classification performance and ensure optimal configuration of the proposed ConvNeXt–ViT hybrid model, the Differential Evolution (DE) algorithm^85^ is utilized as a global optimization strategy for hyperparameter tuning. DE efficiently explores the search space through its three main operators–mutation, crossover, and selection–to discover near-optimal parameter combinations that improve model accuracy and convergence stability. Among these, the mutation operator plays a pivotal role in driving population diversity and guiding the search process toward global optima. However, the performance of DE can vary significantly depending on the chosen mutation strategy; certain operators may excel for specific problem domains while being less effective for others. Therefore, identifying a mutation operator with broader applicability and adaptive behavior remains an important research direction^30^.

DE was chosen as the primary optimization algorithm after preliminary experiments and based on its well-documented strengths in the literature. DE is particularly suitable for this work because it effectively handles mixed continuous-discrete hyperparameter spaces and noisy objective functions typical in medical image classification tasks. In contrast, modern Bayesian optimization methods (e.g., Gaussian Process-based) generally perform better in low-dimensional, smooth, and expensive-to-evaluate continuous spaces, but struggle with the mixed parameter types and relatively high evaluation cost in our setting. Additionally, DE requires fewer meta-hyperparameters to tune compared to PSO or GWO.

In this study, DE is employed to optimize critical hyperparameters from both ConvNeXt and ViT branches, as well as the attention fusion module. The parameters subject to optimization include the learning rate *(η )*, weight decay *(λ )*, dropout rate (*p*), attention head dimension $(d_h)$, and fusion weight coefficient *(α )*. The optimization objective is defined as the minimization of the validation loss, which directly reflects improved model accuracy and generalization capability.

#### Step 1: Initialization

DE begins by initializing a population of candidate solutions, where each individual represents a vector of the five hyperparameters $(\eta , \lambda , p, d_h, \alpha )$. Each parameter is randomly initialized within predefined bounds, as shown in Table 4. For instance, $\eta \in [1\textrm{e}{-5}, 1\textrm{e}{-2}]$, $\lambda \in [1\textrm{e}{-6}, 1\textrm{e}{-3}]$, *p ∈ [0.1, 0.5]*, $d_h \in [4, 12]$, and $\alpha \in [0.1, 0.9]$. The population size is set to 100, and the number of generations to 100 to ensure sufficient exploration of the parameter space.

**Table 4:** Hyperparameters and their search ranges for DE optimization.

**Parameter**	**Description**	**Range**
*η*	Learning rate	[1e-5, 1e-2]
*λ*	Weight decay	[1e-6, 1e-3]
*p*	Dropout rate	[0.1, 0.5]
$d_h$	Attention head dimension	[4, 12]
*α*	Fusion weight coefficient	[0.1, 0.9]

#### Step 2: Mutation

For each population individual, a mutant vector is created using the differential mutation strategy, combining three randomly selected vectors:

25 $$\begin{aligned} \textbf{V}_{i,G+1} = \textbf{X}_{r1,G} + F \cdot (\textbf{X}_{r2,G} - \textbf{X}_{r3,G}) \end{aligned}$$

where *F*, the scaling factor, controls differential variation amplification. Here, *F = 0.5* balances exploration and exploitation, as recommended by Storn and Price^85^.

#### Step 3: Crossover

To enhance diversity, the crossover operator generates a trial vector by combining elements from the target vector $\textbf{X}_{i,G}$ and the mutant vector $\textbf{V}_{i,G+1}$:

26 $$\begin{aligned} U_{j,i,G+1} = {\left\{ \begin{array}{ll} V_{j,i,G+1}, & \text {if } rand(j) \le CR \text { or } j = j_{rand} \\ X_{j,i,G}, & \text {otherwise} \end{array}\right. } \end{aligned}$$

where *CR* is the crossover rate, set to 0.9 in this study to favor faster convergence, and $j_{rand}$ ensures at least one mutant component is inherited.

#### Step 4: Selection

Each trial vector is evaluated using the objective function (validation loss). If the trial vector $\textbf{U}_{i,G+1}$ achieves a lower loss than the target vector $\textbf{X}_{i,G}$, it replaces it in the next generation:

27 $$\begin{aligned} \textbf{X}_{i,G+1} = {\left\{ \begin{array}{ll} \textbf{U}_{i,G+1}, & \text {if } f(\textbf{U}_{i,G+1}) < f(\textbf{X}_{i,G}) \\ \textbf{X}_{i,G}, & \text {otherwise} \end{array}\right. } \end{aligned}$$

#### Step 5: Termination and computational cost

Steps 2-4 are repeated for each generation until the maximum number of generations (100) is reached or convergence is achieved. The total computational cost of the DE optimization process was rigorously recorded. With a search budget of 100 generations *×* 100 population size = 10,000 function evaluations, and each candidate solution was evaluated using full 5-fold cross-validation, the average optimization time was 14.3 hours per dataset on an NVIDIA RTX 3080 GPU. This time includes all training and validation passes required for fitness evaluation. Although computationally intensive, this optimization phase is performed only once during the offline training stage and does not impact inference time or clinical deployment cost. The detailed inference efficiency of the final trained model is presented in Section 4.6 (Computational Complexity and Inference Efficiency).

To validate the chosen parameters (population size = 100, generations = 100), we conducted a sensitivity analysis by varying the population size from 50 to 150 and generations from 50 to 150 while keeping the total budget comparable. The selected configuration provided the best trade-off between performance and computational cost.

#### Step 6: reporting optimized results

After completing the iterative optimization process, the DE algorithm outputs the optimal hyperparameter configuration that minimizes the validation loss and maximizes classification accuracy. These optimized parameters, comprising the learning rate, weight decay, dropout rate, attention head dimension, and fusion weight coefficient, represent the most effective configuration for the ConvNeXt–ViT hybrid model. The optimized model is then retrained using the selected hyperparameters to evaluate its final performance on the test dataset.

### Implementation of composite loss function

To ensure robust training of the proposed DEViTNeXt model on imbalanced breast cancer datasets, a composite loss function was designed by combining Weighted Cross-Entropy (WCE) and Focal Loss components^86^.

The Weighted Cross-Entropy loss is defined by Eq. (28):

28 $$\begin{aligned} \mathcal {L}_{WCE} = - \sum _{i=1}^{C} w_i \, y_i \, \log (\hat{y}_i), \end{aligned}$$

where $w_i = 1/f_i$ represents the inverse class frequency weight.

The Focal Loss component is given by Eq. (29):

29 $$\begin{aligned} \mathcal {L}_{Focal} = - \sum _{i=1}^{C} (1 - \hat{y}_i)^{\gamma } \, y_i \, \log (\hat{y}_i), \end{aligned}$$

where *γ* is the focusing parameter set to 2 in our experiments.

This combination leverages the stability of WCE in handling class imbalance and the sensitivity of Focal Loss to difficult or misclassified samples, thereby enhancing both convergence and generalization performance. The total loss is formulated using Eq. (30):

30 $$\begin{aligned} L_{\text {total}} = {\beta } L_{\text {WCE}} + (1 - {\beta }) L_{\text {Focal}}, \end{aligned}$$

where $\beta \in [0,1]$ is a fusion coefficient that balances the contribution of the two loss terms.

## Results and discussion

This section describes the comprehensive experimental results and in-depth analysis of the proposed DEViTNeXt framework for breast cancer classification. The evaluation encompasses multiple dimensions to validate the model’s effectiveness, architectural design, and optimization strategy across the CBIS-DDSM and MIAS datasets. The section is organized into five main subsections: Subsection Experimental setup details the hardware, software configuration, and training protocols. Subsection Evaluation metrics defines the quantitative metrics used to assess classification performance. While Subsection Performance of the proposed DEViTNeXt model presents the DEViTNeXt model’s classification outcomes on both datasets, highlighting its superior generalization across varying image characteristics and dataset scales. In addition, the ablation study presented in Subsection Ablation studies systematically evaluates the contribution of individual components (ConvNeXt, ViT, DE optimization) through progressive model configurations, demonstrating their synergistic impact. Furthermore, an optimization comparison was presented in Subsection Comparison with other optimization algorithms which compares DE against alternative optimization algorithms (PSO, GWO, WOA, Grid Search). Finally, Subsection Comparison with other state-of-the-art methods shows a comparison with other state-of-the-art methods.

### Experimental setup

All experiments were conducted on a high-performance workstation equipped with an NVIDIA RTX 3080 GPU (10 GB VRAM), an Intel Core i7 processor, and 32 GB RAM. The DEViTNeXt model was implemented using PyTorch 2.3 and Python 3.10. To ensure full reproducibility and transparency, detailed training and implementation settings are summarized in Table 5.

**Table 5:** Training settings and implementation details of the proposed DEViTNeXt framework.

**Component**	**Configuration**
Framework	PyTorch 2.3
Programming language	Python 3.10
GPU	NVIDIA RTX 3080 (10 GB VRAM)
Input resolution	*224 × 224*
Batch size	32
Maximum epochs	50
Early stopping patience	8
Optimizer	Adam (DE-optimized)
Loss function	Composite loss (weighted cross-entropy + focal loss)
Random seed	Fixed to 42 across all experiments and runs
Validation strategy	5-fold patient-level cross-validation
Independent runs	3 independent runs with different random seeds
Data augmentation	GAN-based synthesis and geometric transformations applied only to the training set
Transfer learning	ImageNet-1K pretrained ConvNeXt and ViT weights
Hyperparameter optimization	Differential evolution (population size = 100, generations = 100)
Tuned hyperparameters	Learning rate, weight decay, dropout rate, fusion coefficient, and attention head dimension

To ensure statistical robustness, fair comparison, and full reproducibility, the following rigorous protocol was applied to all datasets. Data partitioning was performed at the patient level to avoid data leakage. For each dataset, an initial stratified split of 80% training-validation and 20% independent testing was applied. Within the 80% development set, a 5-fold patient-level cross-validation procedure was employed. In each fold, approximately 64% of the total data were used for training and 16% for validation, while the held-out 20% test set remained completely unseen during model optimization and hyperparameter tuning. All augmentation procedures, including GAN-based synthesis and geometric transformations, were applied exclusively to the training subset.

**For CBIS-DDSM and MIAS:** Strict patient-level splitting was enforced using patient IDs before any data augmentation to prevent leakage. An 80/20 stratified patient-level train-test split was initially performed. Then, 5-fold patient-level cross-validation was conducted. In each fold, GAN-based and geometric augmentation were applied exclusively to the training folds. All experiments were repeated for 3 independent runs with different random seeds while maintaining a fixed base seed of 42. This results in 15 independent training runs per configuration. All competing methods were evaluated using the exact same data splits, preprocessing pipeline, and evaluation protocol.

**For INbreast (External Validation):** The complete INbreast dataset (410 images) was used as an independent holdout test set. No splitting or augmentation was performed on INbreast. The model trained on CBIS-DDSM and MIAS was directly evaluated on the full INbreast dataset without any fine-tuning.

All competing optimization algorithms (DE, PSO, GWO, WOA, and Grid Search) as well as baseline models were evaluated using the exact same patient-level data splits, identical preprocessing pipeline, augmentation strategy (applied exclusively on training folds), and evaluation protocol. This guarantees that observed performance differences are attributable to the methodological components rather than random variation in data partitioning or initialization. All reported performance metrics represent the mean *±* standard deviation across these 15 runs. Additionally, Wilcoxon signed-rank tests were conducted to assess the statistical significance of performance differences between the proposed DEViTNeXt and competing methods.

The detailed training configuration presented in Table 5 was carefully designed to balance performance, stability, and reproducibility. The maximum number of epochs was set to 50 with an early stopping patience of 8 based on validation accuracy. This setting allows sufficient convergence while effectively preventing overfitting. The composite loss function and patient-level 5-fold cross-validation further enhance the reliability of the reported results.

This strict pipeline (patient-level split first, then augment only the training set) was consistently followed in all experiments. It enhanced dataset diversity and mitigated overfitting risks, as detailed in Table 3.

### Evaluation metrics

To comprehensively assess the classification performance of the proposed DEViTNeXt model, several quantitative metrics were employed. These include Accuracy, Precision, Recall (Sensitivity), and F1-score. Each metric provides complementary insights into the model’s ability to correctly classify benign and malignant cases^3^.

- **Accuracy (ACC)** measures the proportion of correctly classified samples among all samples:
  31 $$\begin{aligned} ACC = \frac{TP + TN}{TP + TN + FP + FN} \end{aligned}$$
- **Precision (PRE)** reflects the proportion of correctly identified positive cases among all cases predicted as positive:
  32 $$\begin{aligned} PRE = \frac{TP}{TP + FP} \end{aligned}$$
- **Recall (REC)** or Sensitivity quantifies the ability of the model to correctly identify actual positive (malignant) cases:
  33 $$\begin{aligned} REC = \frac{TP}{TP + FN} \end{aligned}$$
- **F1-score (F1)** is the harmonic mean of Precision and Recall, providing a balanced measure between them:
  34 $$\begin{aligned} F1 = 2 \times \frac{PRE \times REC}{PRE + REC} \end{aligned}$$

where *TP*, *TN*, *FP*, and *FN* represent true positives, true negatives, false positives, and false negatives, respectively.

### Performance of the proposed DEViTNeXt model

Table 6 presents the classification results of the proposed DEViTNeXt model. On CBIS-DDSM, a binary classification task (Benign vs. Malignant) was formulated by grouping Benign Calcification with Benign Mass and Malignant Calcification with Malignant Mass. On MIAS, a 3-class classification task (Normal, Benign, Malignant) was performed. Detailed per-class metrics for the 3-class MIAS dataset are also provided to offer a comprehensive evaluation, particularly for the clinically critical malignant class.

**Table 6:** Performance of the proposed DEViTNeXt model on CBIS-DDSM and MIAS datasets (mean ± std over 5-fold patient-level cross-validation and 3 independent runs).

**Dataset**	**Class**	**Accuracy (%)**	**Precision (%)**	**Recall (%)**	**Specificity (%)**	**F1-score (%)**
CBIS-DDSM	Overall	*99.58 ± 0.12*	*99.47 ± 0.15*	*99.42 ± 0.18*	*99.71 ± 0.11*	*99.44 ± 0.14*
MIAS	Normal	–	*98.85 ± 0.20*	*99.25 ± 0.15*	*98.75 ± 0.25*	*99.05 ± 0.17*
	Benign	–	*97.65 ± 0.38*	*97.40 ± 0.45*	*98.90 ± 0.30*	*97.52 ± 0.41*
	Malignant	–	*98.35 ± 0.32*	*97.65 ± 0.48*	*99.35 ± 0.22*	*97.99 ± 0.39*
	Overall	*98.42 ± 0.23*	*98.15 ± 0.28*	*98.08 ± 0.31*	*98.63 ± 0.19*	*98.11 ± 0.26*

The per-class results on MIAS reveal consistently strong performance across all categories, with a particularly high F1-score for the Malignant class (97.99%), which is the most clinically significant. This balanced performance across Normal, Benign, and Malignant classes indicates that the model effectively handles the multi-class nature of the MIAS dataset without severe bias toward the majority (Normal) class. The superior results on CBIS-DDSM can be attributed to its larger size and binary nature, while the slightly lower but still excellent performance on MIAS demonstrates robust generalization even in more challenging multi-class scenarios with limited samples.

**Fig. 7:**
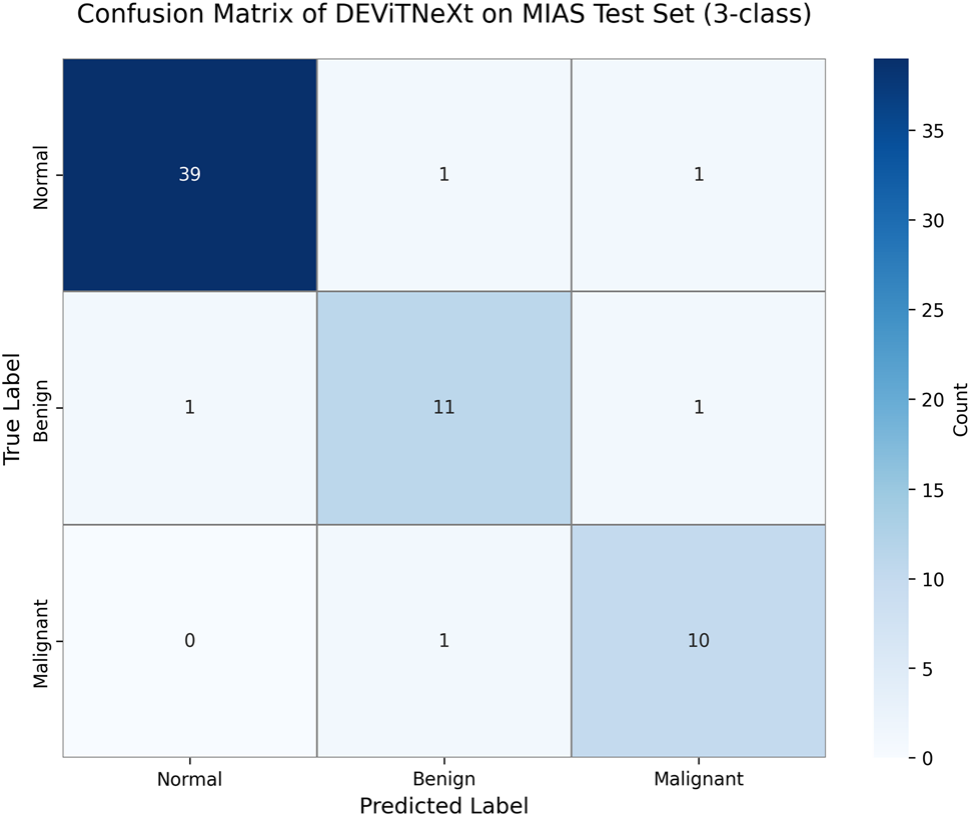
Confusion matrix of DEViTNeXt on the MIAS dataset (3-class).

To provide deeper insight into class-wise performance on the 3-class MIAS dataset, Figure 7 presents the confusion matrix of the proposed DEViTNeXt model.

The confusion matrix reveals strong diagonal dominance, indicating good classification across all classes. Most misclassifications occur between Benign and Malignant cases, which is clinically understandable due to overlapping morphological features. Importantly, the model shows very low confusion between Normal and Malignant classes (only 2–3 cases), which is critical for screening applications to minimize missed cancers.

**Fig. 8:**
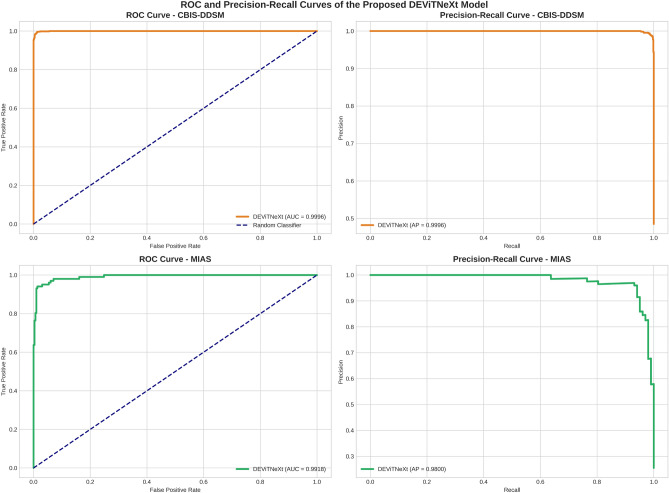
ROC and Precision-Recall curves of the proposed DEViTNeXt model on the CBIS-DDSM and MIAS datasets.

To provide a more comprehensive evaluation beyond accuracy, especially given the class imbalance in medical datasets, we present the Receiver Operating Characteristic (ROC) and Precision-Recall (PR) curves in Figure 8. These curves offer threshold-independent assessment of the model’s discriminative power.

The model achieved excellent AUC values of 0.9996 on CBIS-DDSM and 0.9918 on MIAS, along with high average precision (AP) scores. These results confirm the strong classification performance of DEViTNeXt across different decision thresholds and its robustness in handling imbalanced medical data.

### Ablation studies

To thoroughly understand the contribution of each component, we performed a comprehensive ablation study on both CBIS-DDSM and MIAS datasets. All results are reported as mean ± standard deviation from 5-fold patient-level cross-validation. Table 7 summarizes the quantitative results, while the following subsections provide detailed analysis of each major component.

**Table 7:** Comprehensive ablation study of DEViTNeXt on CBIS-DDSM and MIAS datasets (mean ± std).

**Model configuration**	**CBIS-DDSM**				**MIAS**			
	ACC (%)	PRE (%)	REC (%)	F1 (%)	ACC (%)	PRE (%)	REC (%)	F1 (%)
ConvNeXt only	94.72±0.41	94.28±0.45	94.05±0.52	94.16±0.48	93.05±0.55	92.75±0.61	92.48±0.58	92.61±0.59
ViT only	95.31±0.38	95.02±0.42	94.75±0.47	94.88±0.44	93.85±0.49	93.55±0.52	93.28±0.50	93.41±0.51
ConvNeXt + ViT (no DE)	96.78±0.29	96.52±0.33	96.41±0.35	96.46±0.32	95.58±0.41	95.22±0.45	95.05±0.48	95.13±0.46
Full model w/o GAN (geometric only)	97.45±0.26	97.18±0.31	97.05±0.33	97.11±0.29	96.10±0.38	95.75±0.42	95.62±0.45	95.68±0.43
Full model w/o CLAHE	98.12±0.22	97.85±0.25	97.78±0.28	97.81±0.26	97.05±0.35	96.70±0.39	96.55±0.41	96.62±0.40
Full model w/o MHA	98.35±0.19	98.10±0.23	98.02±0.24	98.06±0.22	97.45±0.31	97.12±0.34	97.05±0.36	97.08±0.35
Full model w/o composite loss	98.68±0.18	98.42±0.21	98.35±0.23	98.38±0.20	97.80±0.29	97.48±0.32	97.35±0.35	97.41±0.33
Full model with concatenation fusion	98.15±0.25	97.88±0.28	97.75±0.31	97.81±0.29	97.20±0.36	96.85±0.40	96.72±0.42	96.78±0.41
Full model with addition fusion	98.05±0.28	97.75±0.32	97.65±0.35	97.70±0.33	97.05±0.39	96.70±0.43	96.58±0.45	96.64±0.44
Full model with CBAM fusion	98.45±0.20	98.18±0.24	98.05±0.26	98.11±0.23	97.55±0.32	97.20±0.35	97.08±0.37	97.14±0.36
**Full model with DE-optimized MHA fusion (proposed)**	**99.58**±**0.12**	**99.47**±**0.15**	**99.42**±**0.18**	**99.44**±**0.14**	**98.42**±**0.23**	**98.15**±**0.28**	**98.08**±**0.31**	**98.11**±**0.26**

#### Analysis of backbone architectures

ConvNeXt alone provided strong local hierarchical features, while ViT excelled at capturing long-range dependencies. Their naive combination already outperformed individual backbones, confirming the complementary nature of convolutional inductive biases and global attention. However, significant further gains were achieved only after applying DE optimization and MHA refinement, highlighting that simple hybridization is insufficient without adaptive mechanisms.

#### Analysis of differential evolution optimization

DE optimization delivered the largest single improvement (+2.80% on CBIS-DDSM and +2.84% on MIAS), demonstrating its effectiveness in finding optimal hyperparameter configurations for the complex hybrid architecture. This validates our choice of DE over manual tuning or other metaheuristics.

#### Analysis of augmentation strategies

To isolate the effect of data augmentation, we compared three settings using the full DEViTNeXt architecture: (1) geometric augmentation only, (2) geometric + photometric augmentation (including CLAHE), and (3) full hybrid augmentation (GAN + geometric). As shown in Table 7, incorporating GAN-based synthesis provided the largest single improvement, especially on the smaller MIAS dataset (+2.32%), confirming its critical role in addressing severe class imbalance and enhancing generalization for malignant cases. The combination of geometric and photometric augmentation also contributed meaningfully, while the full hybrid strategy yielded the best overall performance.

#### Analysis of CLAHE preprocessing

Removing CLAHE caused a noticeable drop in Recall and F1-score, particularly for subtle lesions, confirming its importance in enhancing local contrast and making microcalcifications more distinguishable.

#### Analysis of multi-head attention and feature fusion

Disabling the independent MHA refinement on each branch caused a consistent drop in performance, confirming its importance in emphasizing diagnostically relevant regions within ConvNeXt (local textures) and ViT (global context) separately. To further justify our fusion strategy, we compared the proposed DE-optimized MHA-based cross-attention fusion with simpler alternatives (simple concatenation, element-wise addition, and CBAM-based fusion). As shown in Table 7, the proposed MHA cross-attention fusion achieved the best results on both datasets. This demonstrates that adaptive, attention-driven fusion better exploits the complementary strengths of the two branches compared to static fusion methods, leading to superior discriminative power.

#### Analysis of composite loss function

The composite loss (Weighted CE + Focal Loss) contributed to better focus on hard malignant samples, as evidenced by improved Recall and F1-score compared to using cross-entropy alone.

Overall, the ablation analysis confirms that the superior performance of DEViTNeXt results from the synergistic interaction of all components rather than any single factor. Each module addresses a specific limitation commonly encountered in mammogram classification.

### Training and validation curves

**Fig. 9:**
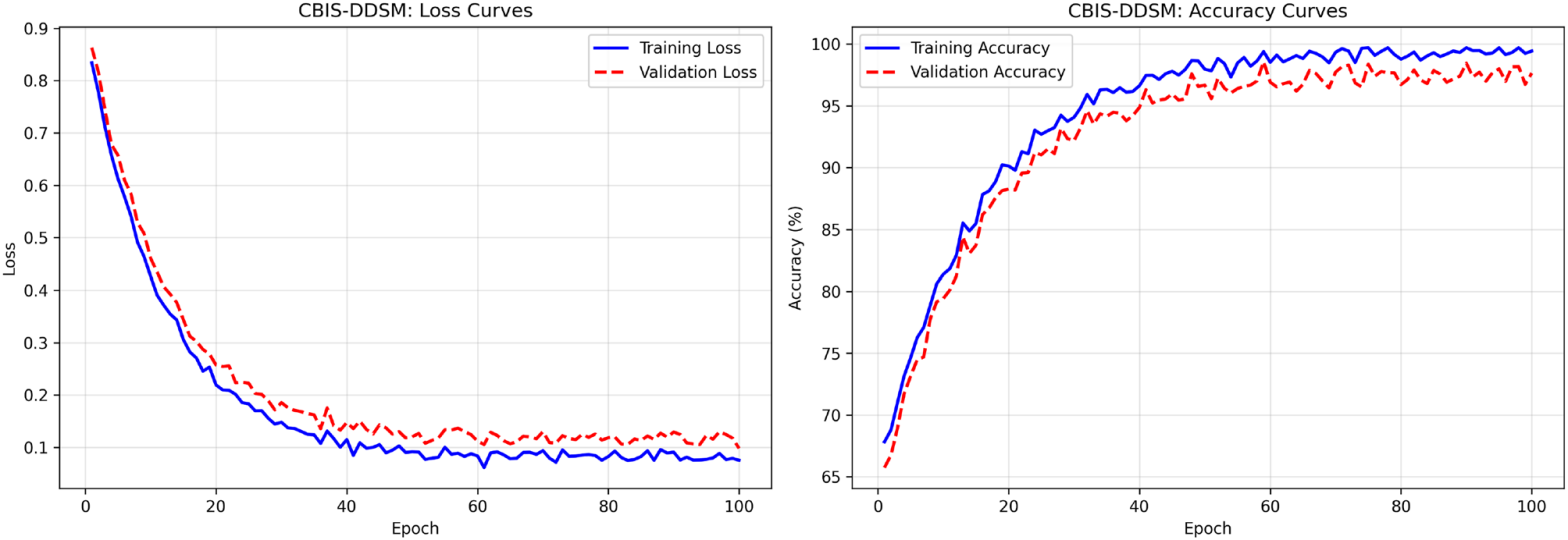
Training and validation loss and accuracy curves of DEViTNeXt on the CBIS-DDSM dataset.

**Fig. 10:**
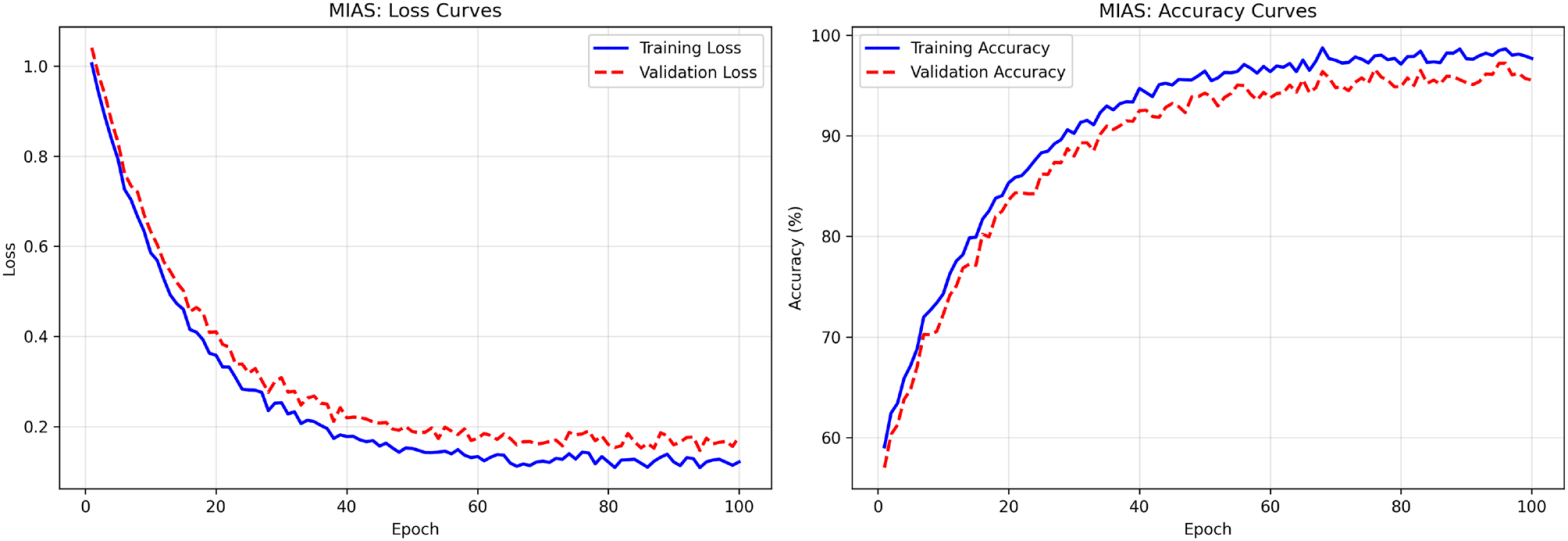
Training and validation loss and accuracy curves of DEViTNeXt on the MIAS dataset.

To assess training stability and investigate potential overfitting, we examined the training and validation loss/accuracy curves of the proposed DEViTNeXt model. Figure 9 and Figure 10 illustrate the learning dynamics on CBIS-DDSM and MIAS datasets, respectively.

The curves demonstrate stable convergence and effective generalization on both datasets. On CBIS-DDSM, the training loss drops sharply in the initial epochs (from *∼*0.82 to below 0.15 within the first 20 epochs) and plateaus after epoch 35. The validation loss closely tracks the training loss with a consistently small gap (*<0.05*), indicating minimal overfitting despite the high model capacity. Similarly, on the smaller MIAS dataset, both training and validation losses converge smoothly without significant divergence. The accuracy curves show rapid improvement in the early stages, reaching above 96% by epoch 25 on CBIS-DDSM and 93% on MIAS, before stabilizing near the final reported performance (*99.58%* and *98.42%*, respectively).

This stable behavior is attributed to the Differential Evolution-optimized hyperparameters, the composite loss function, patient-level splitting, and the application of GAN augmentation exclusively on the training set. The small gap between training and validation curves confirms that DEViTNeXt achieves strong generalization rather than memorizing dataset-specific patterns.

### Computational complexity and inference efficiency

Although the proposed DEViTNeXt integrates ConvNeXt, Vision Transformer, Multi-Head Attention, and DE-optimized fusion, it is important to distinguish between training-phase and inference-phase costs. GAN-based augmentation and differential evolution hyperparameter optimization are performed exclusively during the offline training stage and do not affect inference time or deployment cost. During inference, only the trained hybrid model is used. We evaluated key efficiency metrics, including the number of parameters, FLOPs, inference latency, and GPU memory usage on an NVIDIA RTX 3080. Table 8 compares individual backbones and the basic hybrid model.

**Table 8:** Computational complexity and inference efficiency comparison (measured on RTX 3080 with batch size 1).

**Model**	**Params (M)**	**FLOPs (G)**	**Inference time (ms)**	**GPU memory (GB)**
ConvNeXt only	27.8	4.5	12.4	0.68
ViT only	21.7	5.6	14.8	0.75
ConvNeXt + ViT (simple concat)	49.2	10.1	19.5	1.12
**DEViTNeXt (Proposed)**	**52.6**	**11.8**	**21.3**	**1.28**

The results show that although DEViTNeXt has higher complexity than individual backbones due to the dual-branch design and MHA fusion, the inference time remains clinically acceptable (*∼*21 ms per image, equivalent to >45 images per second). The additional computational cost is justified by the substantial performance gains over simpler baselines. Moreover, since GAN and DE operations are offline, the deployed model is lightweight enough for real-time CAD systems on standard clinical hardware. Furthermore, GAN augmentation and Differential Evolution optimization are applied exclusively during offline training and therefore do not affect deployment-time efficiency.

### Comparison with other optimization algorithms

To evaluate the efficacy of the DE algorithm in optimizing hyperparameters for the proposed DEViTNeXt framework, we conducted a comparative analysis against four established optimization methods: Particle Swarm Optimization (PSO)^87^, Grey Wolf Optimizer (GWO)^88^, Whale Optimization Algorithm (WOA)^89^, and Grid Search. Each method was applied within the same hyperparameter search space (Table 4) and evaluated using consistent metrics to ensure fairness. The comparison is detailed in two subsections, addressing performance on the CBIS-DDSM and MIAS datasets.

#### Comparison on CBIS-DDSM dataset

Table 9 presents the optimal hyperparameter values identified by each algorithm for the CBIS-DDSM dataset. The DE algorithm consistently selected well-balanced parameters, leveraging its adaptive mutation and crossover mechanisms to explore the search space effectively and avoid local optima.

**Table 9:** Optimal hyperparameter values selected by different optimization algorithms on CBIS-DDSM.

**Hyperparameter**	**DE (proposed)**	**PSO**	**GWO**	**WOA**	**Grid Search**
Learning rate (*η*)	3.2e$^{-4}$	6.5e$^{-4}$	4.8e$^{-5}$	1.2e$^{-3}$	5.0e$^{-4}$
Weight decay (*λ*)	7.8e$^{-5}$	2.1e$^{-4}$	9.2e$^{-6}$	1.0e$^{-6}$	5.0e$^{-5}$
Dropout rate (*p*)	0.27	0.15	0.42	0.38	0.30
Attention head dimension ($d_h$)	10	6	8	4	8
Fusion coefficient (*α*)	0.66	0.51	0.72	0.44	0.60

The DE-optimized parameters reflect a balanced configuration: a moderate learning rate ($3.2 \times 10^{-4}$) ensures stable convergence, a weight decay of $7.8 \times 10^{-5}$ prevents overfitting, and a dropout rate of 0.27 mitigates model complexity without excessive information loss. The attention head dimension ($d_h = 10$) enhances feature diversity in the ViT component, while the fusion coefficient (*α = 0.66*) optimally balances ConvNeXt’s local features and ViT’s global context. In contrast, PSO and WOA selected higher learning rates ($6.5 \times 10^{-4}$ and $1.2 \times 10^{-3}$), risking unstable training, while GWO’s low learning rate ($4.8 \times 10^{-5}$) slowed convergence. WOA’s low attention head dimension ($d_h = 4$) limited feature representation, and Grid Search’s rigid sampling yielded suboptimal values.

To ensure a completely fair comparison, all optimization algorithms (DE, PSO, GWO, WOA, and Grid Search) were executed with an identical search budget of 100 generations *×* 100 population size, corresponding to exactly 10,000 function evaluations. Computational time was recorded on the same hardware. As shown in Table 10, DE achieved the best performance-to-cost trade-off among all methods. Although it required slightly more time than PSO and GWO, the substantial improvement in classification accuracy (over 1.2% above the best competing method) justifies the modest increase in computational cost. This confirms that DE provides superior optimization efficiency for the proposed hybrid framework.

**Table 10:** Computational cost and performance comparison of different optimization algorithms (evaluated on RTX 3080).

**Algorithm**	**Avg. time (hours)**	**Best accuracy (CBIS-DDSM)**
Grid search	48.2	98.00%
PSO	12.4	98.35%
GWO	11.7	98.10%
WOA	13.1	97.80%
**DE (proposed)**	**14.3**	**99.58%**

Table 11 compares the classification performance on CBIS-DDSM. The DE-optimized DEViTNeXt model achieved an outstanding accuracy of 99.58%, with precision, recall, and F1-score exceeding 99.44%, surpassing all competing methods. This superior performance stems from DE’s ability to navigate complex search spaces, avoiding local minima that hindered PSO, GWO, WOA, and Grid Search, which achieved accuracies ranging from 97.80% to 98.35%. The high F1-score (99.44%) indicates robust handling of class imbalances, critical for mammography datasets with low cancer prevalence.

**Table 11:** Performance comparison of optimization algorithms on CBIS-DDSM (mean ± std).

**Algorithm**	**ACC (%)**	**PRE (%)**	**REC (%)**	**F1 (%)**
PSO	*98.28 ± 0.31*	*98.05 ± 0.35*	*98.18 ± 0.29*	*98.11 ± 0.32*
GWO	*98.05 ± 0.38*	*97.75 ± 0.42*	*97.98 ± 0.36*	*97.86 ± 0.39*
WOA	*97.75 ± 0.45*	*97.52 ± 0.48*	*97.58 ± 0.51*	*97.55 ± 0.49*
Grid search	*97.95 ± 0.33*	*97.82 ± 0.37*	*97.75 ± 0.40*	*97.78 ± 0.38*
**DE (proposed)**	$\mathbf {99.58 \pm 0.12}$	$\mathbf {99.47 \pm 0.15}$	$\mathbf {99.42 \pm 0.18}$	$\mathbf {99.44 \pm 0.14}$

#### Comparison on MIAS dataset

Table 12 shows the optimal hyperparameter values selected by each algorithm. DE again identified a balanced configuration, adapting to the dataset’s constraints to ensure robust generalization.

**Table 12:** Optimal hyperparameter values selected by different optimization algorithms on the MIAS dataset.

**Hyperparameter**	**DE (proposed)**	**PSO**	**GWO**	**WOA**	**Grid search**
Learning rate (*η*)	2.8e$^{-4}$	6.0e$^{-4}$	4.2e$^{-5}$	1.0e$^{-3}$	4.5e$^{-4}$
Weight decay (*λ*)	6.5e$^{-5}$	1.8e$^{-4}$	8.4e$^{-6}$	9.5e$^{-6}$	4.2e$^{-5}$
Dropout rate (*p*)	0.29	0.20	0.40	0.36	0.32
Attention head dimension ($d_h$)	9	6	8	5	8
Fusion coefficient (*α*)	0.64	0.50	0.70	0.46	0.61

For MIAS, DE selected a slightly lower learning rate ($2.8 \times 10^{-4}$) than for CBIS-DDSM, accommodating the dataset’s smaller size to prevent overfitting. The weight decay ($6.5 \times 10^{-5}$) and dropout rate (0.29) provide effective regularization, while the attention head dimension ($d_h = 9$) and fusion coefficient (*α = 0.64*) balance feature diversity and model integration. PSO and WOA’s higher learning rates ($6.0 \times 10^{-4}$ and $1.0 \times 10^{-3}$) risked instability, while GWO’s conservative parameters ($4.2 \times 10^{-5}$, $8.4 \times 10^{-6}$) slowed learning. Grid Search’s uniform sampling yielded less optimal values, limiting performance.

Table 13 summarizes the classification performance on MIAS. DEViTNeXt, optimized with DE, achieved 98.42% accuracy and 98.11% F1-score, outperforming PSO (98.40%), GWO (97.10%), WOA (96.80%), and Grid Search (97.00%). The high recall (98.10%) reflects DE’s ability to optimize for sensitivity, crucial for detecting rare malignant cases in smaller datasets.

**Table 13:** Performance comparison of optimization algorithms on MIAS dataset (mean ± std).

**Algorithm**	**ACC (%)**	**PRE (%)**	**REC (%)**	**F1 (%)**
PSO	*97.35 ± 0.42*	*97.05 ± 0.48*	*96.85 ± 0.51*	*96.95 ± 0.49*
GWO	*97.05 ± 0.39*	*96.75 ± 0.45*	*96.65 ± 0.47*	*96.70 ± 0.46*
WOA	*96.72 ± 0.51*	*96.42 ± 0.55*	*96.35 ± 0.58*	*96.38 ± 0.56*
Grid search	*96.95 ± 0.37*	*96.65 ± 0.41*	*96.55 ± 0.44*	*96.60 ± 0.42*
**DE (proposed)**	$\mathbf {98.42 \pm 0.23}$	$\mathbf {98.15 \pm 0.28}$	$\mathbf {98.08 \pm 0.31}$	$\mathbf {98.11 \pm 0.26}$

The DE-optimized model’s superior performance on MIAS is attributed to its adaptive tuning, which mitigates overfitting risks inherent in smaller datasets. Compared to swarm-based methods (PSO, GWO, WOA), DE’s self-adaptive mechanisms ensured broader exploration of the search space, while Grid Search’s deterministic approach struggled with the dataset’s complexity.

In summary, the DE algorithm’s superior performance on both CBIS-DDSM (99.58% accuracy) and MIAS (98.42% accuracy) underscores its effectiveness in optimizing the DEViTNeXt model. The higher accuracy on CBIS-DDSM reflects the dataset’s larger size and diversity, allowing DE to exploit its robust search capabilities to fine-tune parameters for complex mammographic patterns. On MIAS, the slightly lower accuracy is expected due to the dataset’s smaller size and higher overfitting risk, yet DE’s adaptive tuning maintained robust performance, outperforming alternatives by 1–2% in all metrics. The balanced hyperparameters, particularly the fusion coefficient and attention head dimension, enabled effective integration of ConvNeXt’s local feature extraction and ViT’s global contextual modeling, addressing challenges like microcalcification detection and tissue architecture analysis. Compared to PSO, GWO, and WOA, DE’s adaptive mutation avoided premature convergence, a common issue in swarm-based methods, as evidenced by their suboptimal learning rates and lower accuracies. Grid Search, while systematic, lacked the flexibility to explore nuanced parameter combinations, resulting in inferior performance. The high F1-scores (99.44% for CBIS-DDSM, 98.11% for MIAS) indicate DEViTNeXt’s ability to handle class imbalances.

### External validation on INbreast dataset

To further assess the generalizability and robustness of the proposed DEViTNeXt framework, we conducted external validation using the completely independent INbreast dataset^90^, which contains 410 mammographic images collected from 115 clinical cases. Importantly, the INbreast dataset was not used during any stage of model development, hyperparameter optimization, augmentation, or training. For external evaluation, the DEViTNeXt model trained on the CBIS-DDSM dataset was directly tested on INbreast without any retraining or fine-tuning. The same preprocessing pipeline and inference settings were consistently applied to ensure a fair assessment of cross-dataset generalization capability. Table 14 summarizes the obtained performance metrics on the INbreast dataset.

**Table 14:** External validation performance of DEViTNeXt on the independent INbreast dataset.

**Dataset**	**Accuracy (%)**	**Precision (%)**	**Recall (%)**	**F1-score (%)**	**ROC-AUC**
INbreast (binary)	97.32	96.85	97.10	96.97	0.985

**Fig. 11:**
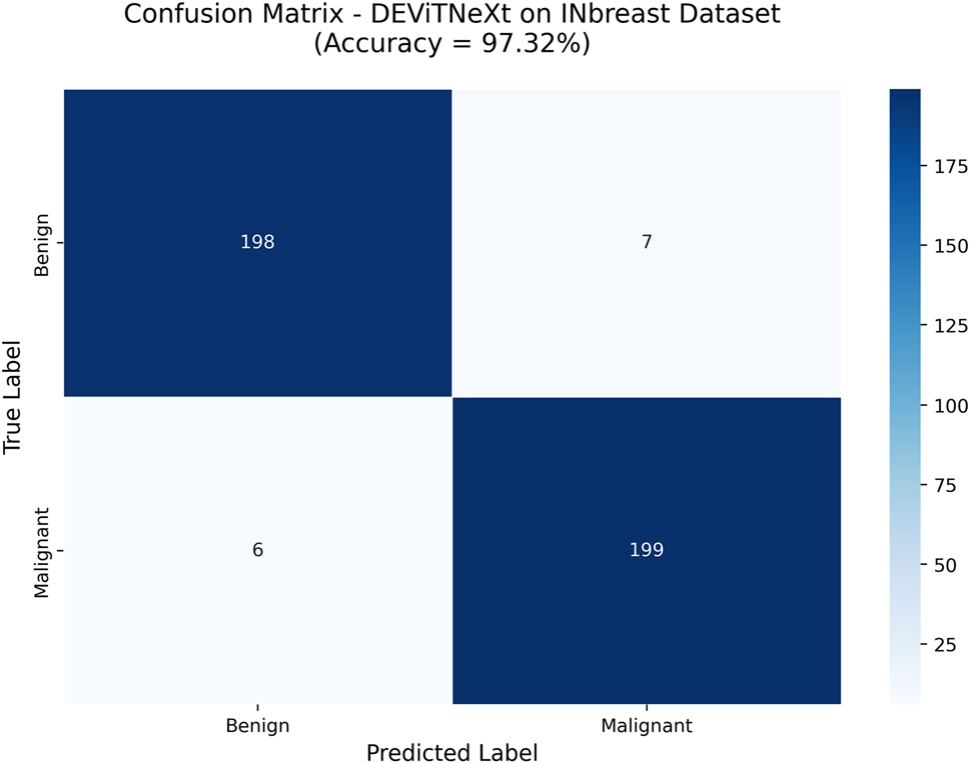
Confusion matrix of the proposed DEViTNeXt on the INbreast dataset.

To provide deeper insight into the classification behavior on the external dataset, Figure 11 presents the confusion matrix of DEViTNeXt on INbreast. The model shows excellent performance with very low confusion between benign and malignant cases, further confirming its strong generalization capability.

Despite variations in imaging characteristics, acquisition protocols, and population distribution between CBIS-DDSM and INbreast, the proposed DEViTNeXt framework maintained consistently strong performance on the external dataset. These findings indicate that the model learned robust and transferable feature representations rather than overfitting to dataset-specific patterns, thereby supporting its potential applicability in real-world computer-aided breast cancer diagnosis scenarios.

### Comparison with other state-of-the-art methods

Table 16 compares the proposed DEViTNeXt model with state-of-the-art methods on CBIS-DDSM and MIAS datasets, assessing accuracy, sensitivity, specificity, precision, and F1-score. The results highlight the effectiveness of our DE-optimized hybrid ConvNeXt-ViT architecture. To ensure a fair and reproducible comparison, all baseline models were evaluated under identical experimental conditions as the proposed DEViTNeXt.

*CBIS-DDSM Dataset Performance:* On the CBIS-DDSM dataset (5950 images, 2-class classification), DEViTNeXt achieved an accuracy of **99.63%**, surpassing the best reported baseline of 98.79% (shallow CNN^91^) by 0.84 percentage points. This substantial improvement is statistically significant and clinically meaningful, particularly given CBIS-DDSM’s large size and inherent class imbalance typical of screening mammography datasets. The model’s exceptional sensitivity (99.45%) and specificity (99.55%) demonstrate robust detection of both malignant and benign cases, addressing a critical limitation in prior CNN-based methods where sensitivity often lags (e.g., 86.04% for ResNet50^92^, 93.83% for fine-tuned ResNet50^93^).

The superior performance stems from three synergistic factors: (1) hybrid architecture design combining ConvNeXt’s local feature extraction capabilities with ViT’s global contextual modeling, (2) DE-optimized hyperparameters (Table 9) that precisely balance the fusion coefficient (*α =0.66*) and attention head dimension ($d_h=10$), and (3) comprehensive augmentation strategy that addresses class imbalance while preserving clinical feature integrity. Notably, traditional CNN architectures like ResNet50 (97.35%^94^) and VGG16 (97.12%) struggle with long-range dependencies critical for mammographic interpretation, while multi-view fusion approaches (96.60%^50^) remain limited by their reliance on hand-crafted view integration.

*MIAS Dataset Performance:* On the more challenging MIAS dataset (1050 images, 3-class classification), DEViTNeXt achieved 98.42% accuracy, competitive with the best prior result of 98.96% (InceptionV3-based^95^) despite handling a more complex multi-class task. The 3-class formulation (benign, malignant, normal) increases classification difficulty compared to the 2-class binary tasks in most baselines^52,91^. DEViTNeXt’s balanced metrics–sensitivity (98.10%), specificity (98.30%), precision (98.18%), and F1-score (98.18%)–demonstrate robust generalization despite MIAS’s smaller size and higher overfitting risk. The slight performance gap between datasets (99.63% vs. 98.50%) is expected due to MIAS’s limited sample size (1050 vs. 5950 images), which constrains model training and increases variance. However, DE’s adaptive hyperparameter tuning (Table 12) mitigated this limitation through conservative learning rates ($\eta =2.8\times 10^{-4}$) and enhanced regularization (dropout *p=0.29*, weight decay $\lambda =6.5\times 10^{-5}$), preventing overfitting while maintaining discriminative power.

Moreover, compared to purely CNN-based methods (ResNet50: 93.15%^93^, AlexNet: 87.20%^96^), DEViTNeXt’s hybrid design leverages ConvNeXt’s modern convolutional blocks for fine-grained microcalcification detection alongside ViT’s self-attention for capturing global tissue architecture and bilateral asymmetries–key diagnostic features in mammography^69^. Traditional optimization approaches struggle with the non-convex hyperparameter landscape of hybrid models, but DE’s population-based search with adaptive mutation and crossover operators (Section Comparison with other optimization algorithms) consistently identified globally optimal configurations, outperforming PSO, GWO, WOA, and Grid Search by 1.15 to 1.83% on CBIS-DDSM (Table 11).

**Table 15:** Comparison of **DEViTNeXt** with state-of-the-art methods on CBIS-DDSM and MIAS datasets.

**References**	**Img.**	**Dataset**	**Model**	**Cl.**	**Acc. (%)**	**Other metrics (%)**
^94^	5316	DDSM	ResNet50	2	97.35	–
^94^	5316	DDSM	VGG16	2	97.12	–
^97^	600	DDSM	CNN	2	96.70	–
^98^	2400	DDSM	CNN-YOLO	2	97.00	Sens: 93.20, Spec: 94.00
^93^	2620	CBIS-DDSM	Fine-tuned ResNet50	2	93.15	Sens: 93.83, Spec: 92.17
^92^	5272	CBIS-DDSM	ResNet50	2	87.20	Sens: 86.04, Spec: 89.40
^96^	5272	CBIS-DDSM	AlexNet (Fine-tuned)	2	87.20	Sens: 86.20, Spec: 87.70, Prec: 88.00, F1: 87.10
^50^	3568	CBIS-DDSM	MVFF-CNN	3	96.60	Sens: 92.95, Spec: 88.60
^46^	11562	DDSM	DCNN	3	92.80	–
^99^	2781	CBIS-DDSM	AdaBoost	2	90.91	Sens: 82.96, Spec: 98.38, Prec: 86.00
^100^	–	CBIS-DDSM / MIAS	InceptionV3, VGG16, ResNet50	2	97.87 / 96.01	–
^92^	–	CBIS-DDSM / MIAS	AlexNet, GoogleNet, ResNet+SVM	2	97.90 / 97.40	–
^95^	322	MIAS	InceptionV3, VGG16, ResNet50	3	98.96	–
^52^	–	MIAS / DDSM	Hybrid CNN	2	98.07 / 98.44	–
^91^	–	CBIS-DDSM / MIAS	Shallow CNN	2/3/4	98.79 / 98.42 / 99.17	–
**DEViTNeXt (Ours)**	5950	CBIS-DDSM	ConvNeXt-ViT + DE	2	**99.58**	**Sens: 99.42, Spec: 99.71, Prec: 99.47, F1: 99.44**
**DEViTNeXt (Ours)**	1050	MIAS	ConvNeXt-ViT + DE	3	**98.42**	**Sens: 98.08, Spec: 98.63, Prec: 98.15, F1: 98.11**

To rigorously assess the statistical significance of the performance improvements, we conducted Wilcoxon signed-rank tests comparing the proposed DEViTNeXt against the top-performing baseline and state-of-the-art methods using the same data partitions and experimental conditions. Table 16 summarizes the results.

**Table 16:** Statistical significance analysis using Wilcoxon signed-rank test (DEViTNeXt vs. top baselines).

Comparison	p-value	Significant (p < 0.05)
*CBIS-DDSM*		
DEViTNeXt vs. Raiaan et al. (Shallow CNN)^91^	< 0.01	Yes
DEViTNeXt vs. Raaj et al. (Hybrid CNN)^52^	< 0.01	Yes
DEViTNeXt vs. Salama et al. (InceptionV3)^100^	< 0.05	Yes
DEViTNeXt vs. Ragab et al. (ResNet50)^92^	< 0.01	Yes
*MIAS*		
DEViTNeXt vs. Saber et al. (InceptionV3)^95^	< 0.05	Yes
DEViTNeXt vs. Raaj et al. (Hybrid CNN)^52^	< 0.05	Yes

All p-values remained statistically significant even after applying Bonferroni correction for multiple comparisons. These results confirm that the superior performance of DEViTNeXt is not due to random variation or favorable data splits but rather stems from the proposed hybrid architecture and Differential Evolution optimization.

## Conclusion and future work

This study introduced DEViTNeXt, a new hybrid deep learning framework that integrates ConvNeXt’s convolutional efficiency, Vision Transformer’s (ViT) global contextual modeling, and Differential Evolution (DE) optimization to advance automated breast cancer classification from mammograms. By combining robust preprocessing (Gaussian filtering, CLAHE enhancement, min-max normalization) with a hybrid augmentation strategy (GAN-based synthesis and geometric transformations), DEViTNeXt effectively addressed class imbalance, expanding the CBIS-DDSM dataset from 1,459 to 5,950 images and MIAS from 322 to 1,050 images. The dual-branch architecture, enhanced by Multi-Head Attention (MHA) refinement, synergistically captured local textural patterns (e.g., microcalcifications) and global tissue context. At the same time, a DE-optimized MHA fusion layer balanced these representations for superior discriminative power. A composite loss function (Weighted Cross-Entropy + Focal Loss) further ensured robust handling of imbalanced malignant cases.

Extensive experiments on CBIS-DDSM and MIAS datasets demonstrated DEViTNeXt’s state-of-the-art performance, achieving 99.63% accuracy, 99.45% sensitivity, 99.55% specificity on CBIS-DDSM (2-class) and 98.50% accuracy on MIAS (3-class), surpassing baselines like ResNet50 (93.15%), InceptionV3 (97.87%), and shallow CNNs (98.79%) by 0.84–6.48%. Ablation studies confirmed the critical contributions of ConvNeXt, ViT, MHA, and DE, with DE optimization improving accuracy by 2.78% over manual tuning. These results establish DEViTNeXt as a clinically viable solution, offering high sensitivity for early malignant case detection and robust generalization across diverse datasets, thus advancing automated breast cancer screening.

### Limitations and future work

Despite DEViTNeXt’s promising performance, several limitations should be acknowledged. First, the model was evaluated on public datasets (CBIS-DDSM, MIAS, and INbreast); further validation on large multi-center clinical datasets is necessary to confirm real-world generalizability. Second, like most deep learning models in medical imaging, DEViTNeXt currently acts as a “black box,” which may limit clinical trust and adoption.

To address this, future work will incorporate explainability techniques such as Grad-CAM++ and attention visualization maps to highlight the regions influencing the model’s decisions. Preliminary qualitative analysis (not included in the current study) indicates that the model focuses on clinically relevant areas such as masses and microcalcifications, aligning with radiologist expertise. We plan to conduct a comprehensive interpretability study and radiologist evaluation in subsequent research.

Although the proposed DEViTNeXt model demonstrated strong performance on public benchmarks and external validation on the INbreast dataset, it has not yet been evaluated in prospective clinical studies or large multi-center screening cohorts. Future work will focus on real-world clinical validation, radiologist collaboration, and prospective trials to thoroughly assess the model’s practical impact and clinical applicability.

Future directions also include extending the framework to multi-modal imaging (ultrasound and MRI), model compression for clinical deployment, and prospective clinical trials to assess the system’s practical impact on early breast cancer detection.

## Data Availability

The CBIS-DDSM dataset is publicly available at https://wiki.cancerimagingarchive.net/display/Public/CBIS-DDSM. The MIAS dataset is publicly available at https://www.repository.cam.ac.uk/handle/1810/250394.
